# Social Media Use and Mental Health and Well-Being Among Adolescents – A Scoping Review

**DOI:** 10.3389/fpsyg.2020.01949

**Published:** 2020-08-14

**Authors:** Viktor Schønning, Gunnhild Johnsen Hjetland, Leif Edvard Aarø, Jens Christoffer Skogen

**Affiliations:** ^1^Department of Health Promotion, Norwegian Institute of Public Health, Bergen, Norway; ^2^Alcohol and Drug Research Western Norway, Stavanger University Hospital, Stavanger, Norway; ^3^Faculty of Health Sciences, University of Stavanger, Stavanger, Norway

**Keywords:** scoping review, social media, mental health, adolescence, well-being

## Abstract

**Introduction:** Social media has become an integrated part of daily life, with an estimated 3 billion social media users worldwide. Adolescents and young adults are the most active users of social media. Research on social media has grown rapidly, with the potential association of social media use and mental health and well-being becoming a polarized and much-studied subject. The current body of knowledge on this theme is complex and difficult-to-follow. The current paper presents a scoping review of the published literature in the research field of social media use and its association with mental health and well-being among adolescents.

**Methods and Analysis:** First, relevant databases were searched for eligible studies with a vast range of relevant search terms for social media use and mental health and well-being over the past five years. Identified studies were screened thoroughly and included or excluded based on prior established criteria. Data from the included studies were extracted and summarized according to the previously published study protocol.

**Results:** Among the 79 studies that met our inclusion criteria, the vast majority (94%) were quantitative, with a cross-sectional design (57%) being the most common study design. Several studies focused on different aspects of mental health, with depression (29%) being the most studied aspect. Almost half of the included studies focused on use of non-specified social network sites (43%). Of specified social media, Facebook (39%) was the most studied social network site. The most used approach to measuring social media use was frequency and duration (56%). Participants of both genders were included in most studies (92%) but seldom examined as an explanatory variable. 77% of the included studies had social media use as the independent variable.

**Conclusion:** The findings from the current scoping review revealed that about 3/4 of the included studies focused on social media and some aspect of pathology. Focus on the potential association between social media use and positive outcomes seems to be rarer in the current literature. Amongst the included studies, few separated between different forms of (inter)actions on social media, which are likely to be differentially associated with mental health and well-being outcomes.

## Background

In just a few decades, the use of social media have permeated most areas of our society. For adolescents, social media play a particularly large part in their lives as indicated by their extensive use of several different social media platforms (Ofcom, 2018). Furthermore, the use of social media and types of platforms offered have increased at such a speed that there is reason to believe that scientific knowledge about social media in relation to adolescents’ health and well-being is scattered and incomplete (Orben, 2020). Nevertheless, research findings indicating the potential negative effects of social media on mental health and well-being are frequently reported in traditional media (newspapers, radio, TV) (Bell et al., 2015). Within the scientific community, however, there are ongoing debates regarding the impact and relevance of social media in relation to mental health and well-being. For instance, Twenge and Campbell (2019) stated that use of digital technology and social media have a negative impact on well-being, while Orben and Przybylski (2019) argued that the association between digital technology use and adolescent well-being is so small that it is more or less inconsequential. Research on social media use is a new focus area, and it is therefore important to get an overview of the studies performed to date, and describe the subject matter studies have investigated in relation to the effect of social media use on adolescents mental health and well-being. Also, research gaps in this emerging research field is important to highlight as it may guide future research in new and meritorious directions. A scoping review is therefore deemed necessary to provide a foundation for further research, which in time will provide a knowledge base for policymaking and service delivery.

This scoping review will help provide an overall understanding of the main foci of research within the field of social media and mental health and well-being among adolescents, as well as the type of data sources and research instruments used so far. Furthermore, we aim to highlight potential gaps in the research literature (Arksey and O’Malley, 2005). Even though a large number of studies on social media use and mental health with different vantage points has been conducted over the last decade, we are not aware of any broad-sweeping scoping review covering this area.

## Aims

This scoping review aims to give an overview of the main research questions that have been focused on with regard to use of social media among adolescents in relation to mental health and well-being. Both quantitative and qualitative studies are of interest. Three specific secondary research questions will be addressed and together with the main research question serve as a template for organizing the results:

• Which aspects of mental health and well-being have been the focus or foci of research so far?
• Has the research focused on different research aims across gender, ethnicity, socio-economic status, geographic location? What kind of findings are reported across these groups?
• Organize and describe the main sources of evidence related to social media that have been used in the studies identified.

## Defining Adolescence and Social Media

In the present review, adolescents are defined as those between 13 and 19 years of age. We chose the mean age of 13 as our lower limit as nearly all social media services require users to be at least 13 years of age to access and use their services (Childnet International, 2018). All pertinent studies which present results relevant for this age range is within the scope of this review. For social media we used the following definition by Kietzmann et al. (2011, p. 1): “Social media employ mobile and web-based technologies to create highly interactive platforms via which individuals and communities share, co-create, discuss, and modify user-generated content.” We also employed the typology described by Kaplan and Haenlein’s classification scheme across two axes: level of self-presentation and social presence/media richness (Kaplan and Haenlein, 2010). The current scoping review adheres to guidelines and recommendations stated by Tricco et al. (2018).

See protocol for further details about the definitions used (Schønning et al., 2020).

## Data Sources and Search Strategy

A literature search was performed in OVID Medline, OVID Embase, OVID PsycINFO, Sociological Abstracts (proquest), Social Services Abstracts (proquest), ERIC (proquest), and CINAHL. The search strategy combined search terms for adolescents, social media and mental health or wellbeing. The database-controlled vocabulary was used for searching subject headings, and a large spectrum of synonyms with appropriate truncations was used for searching title, abstract, and author keywords. A filter for observational studies was applied to limit the results. The search was also limited to publications from 2014 to current. The search strategy was translated between each database. An example of full strategy for Embase is attached as Supplementary Material.

## Study Selection: Exclusion and Inclusion Criteria

The exclusion and inclusion criteria are detailed in the protocol (Schønning et al., 2020). Briefly, we included English language peer-reviewed quantitative- or qualitative papers or systematic reviews published within the last 5 years with an explicit focus on mental health/well-being and social media. Non-empirical studies, intervention studies, clinical studies and publications not peer-reviewed were excluded. Intervention studies and clinical studies were excluded as we sought to not introduce too much heterogeneity in design and our focus was on observational studies. The criteria used for study selection was part of an iterative process which was described in detail in the protocol (Schønning et al., 2020). As per the study protocol (Schønning et al., 2020), and in line with scoping review guidelines (Peters et al., 2015, 2017; Tricco et al., 2018), we did not assess methodological quality or risk of bias of the included studies.

**FIGURE 1:**
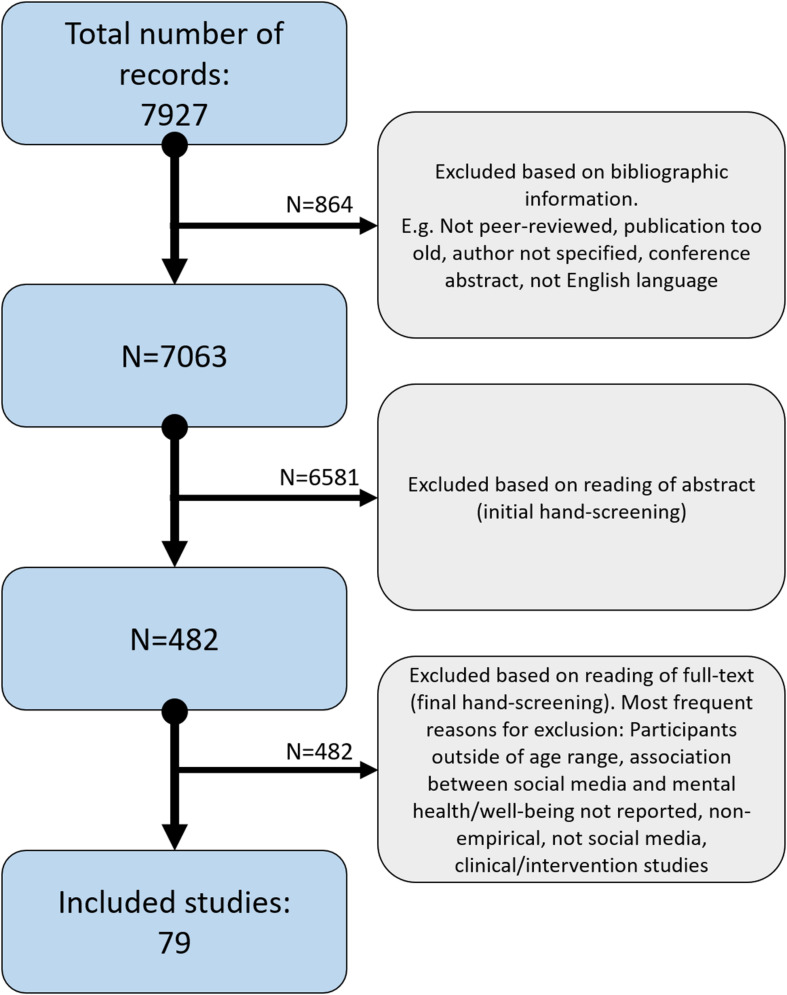
Flowchart of exclusion process from unsorted results to included studies.

The selection process is illustrated by a flow-chart indicating the stages from unsorted search results to the number of included studies (see Figure 1). Study selection was accomplished and organized using the Rayyan QCRI software^1^. The inclusion and exclusion process was performed independently by VS and JCS. The interrater agreement was κ = 0.87, indicating satisfactory agreement.

## Data Extraction and Organization

Details of the data extracted is described in the protocol. Three types of information were extracted, bibliographic information, information about study design and subject matter information. Subject matter information included aim of study, how social media and mental health/well-being was measured, and main findings of the study.

### Visualization of Words From the Titles of the Included Studies

**FIGURE 2:**
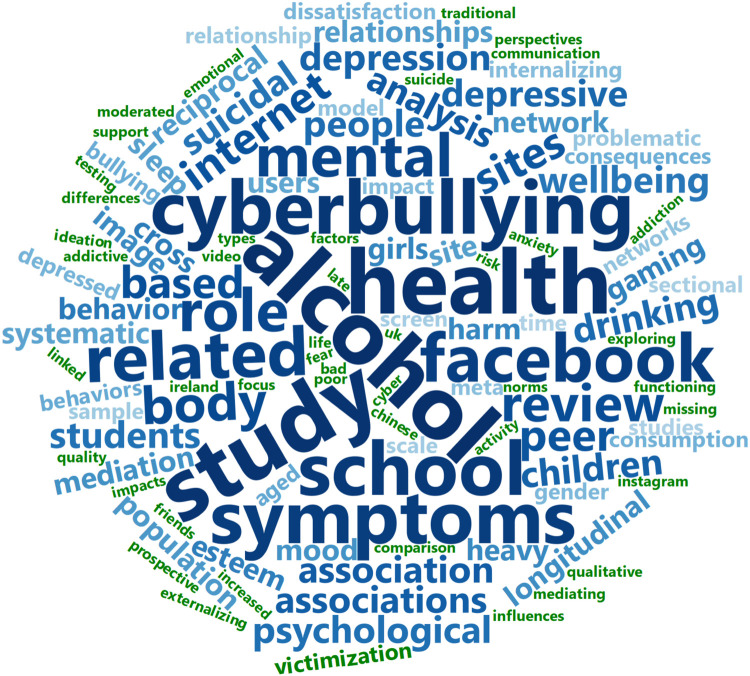
Word cloud from the titles of the included studies. Most frequent words, excluding variations of “adolescence” and “social media.” *N* = 113. Shades of blue indicate word frequencies >2 and green a frequency of 2. The size of each word is indicative of its relative frequency of occurrence.

**FIGURE 3:**
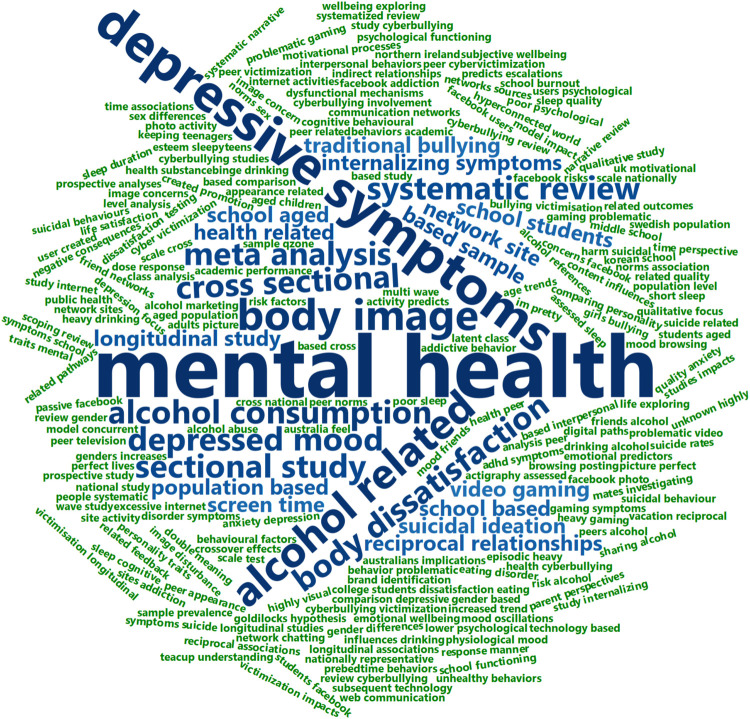
Word cloud from the titles of the included studies. Bigrams from the titles of the included studies, excluding variations of “adolescence” and “social media.” *N* = 231. Shades of blue indicate bigram frequencies of >1 and green a frequency of 1. The size of each bigram is indicative of its relative frequency of occurrence.

The most frequently occurring words and bigrams in the titles of the included studies are presented in Figures 2, 3. The following procedure was used to generate Figure 1: First, a text file containing all titles were imported into R as a data frame (R Core Team, 2014). The data frame was processed using the “tidy text”-package with required additional packages (Silge and Robinson, 2016). Second, numbers and commonly used words with little inherent meaning (so called “stop words,” such as “and,” “of,” and “in”), were removed from the data frame using the three available lexicons in the “tidy-text”-package (Silge and Robinson, 2016). Furthermore, variations of “adolescents” (e.g., “adolescent,” “adolescence,” and “adolescents”) and “social media” (e.g., “social media,” “social networking,” “online social networks”) were removed from the data frame. Third, the resulting data frame was sorted based on frequency of unique words, and words occurring only once were removed. The final data frame is presented as a word cloud in Figure 1 (*N* = 113). The same procedure as described above was employed to generate commonly occurring bigrams (two words occurring adjacent to each other), but without removing bigrams occurring only once (*N* = 231). The word clouds were generated using the “wordcloud2”-package in R (Lang and Chien, 2018). For Figure 1, shades of blue indicate word frequencies >2 and green a frequency of 2. For Figure 2, shades of blue indicate bigram frequencies of >1 and green a frequency of 1.

## Results

### Characteristics of the Included Studies

Of 7927 unique studies, 79 (1%) met our inclusion criteria (Aboujaoude et al., 2015; Banjanin et al., 2015; Banyai et al., 2017; Barry et al., 2017; Best et al., 2014, 2015; Booker et al., 2018; Bourgeois et al., 2014; Boyle et al., 2016; Brunborg et al., 2017; Burnette et al., 2017; Colder Carras et al., 2017; Critchlow et al., 2019; Cross et al., 2015; Curtis et al., 2018; de Lenne et al., 2018; de Vries et al., 2016; Erfani and Abedin, 2018; Erreygers et al., 2018; Fahy et al., 2016; Ferguson et al., 2014; Fisher et al., 2016; Foerster and Roosli, 2017; Foody et al., 2017; Fredrick and Demaray, 2018; Frison and Eggermont, 2016, 2017; Geusens and Beullens, 2017, 2018; Hamm et al., 2015; Hanprathet et al., 2015; Harbard et al., 2016; Hase et al., 2015; Holfeld and Mishna, 2019; Houghton et al., 2018; Jafarpour et al., 2017; John et al., 2018; Kim et al., 2019; Kim, 2017; Koo et al., 2015; Lai et al., 2018; Larm et al., 2017, 2019; Marchant et al., 2017; Marengo et al., 2018; Marques et al., 2018; Meier and Gray, 2014; Memon et al., 2018; Merelle et al., 2017; Neira and Barber, 2014; Nesi et al., 2017a, b; Niu et al., 2018; Nursalam et al., 2018; Oberst et al., 2017; O’Connor et al., 2014; O’Reilly et al., 2018; Przybylski and Bowes, 2017; Przybylski and Weinstein, 2017; Richards et al., 2015; Rousseau et al., 2017; Salmela-Aro et al., 2017; Sampasa-Kanyinga and Chaput, 2016; Sampasa-Kanyinga and Lewis, 2015; Sampasa-Kanyinga et al., 2018; Scott and Woods, 2018; Settanni et al., 2018; Spears et al., 2015; Throuvala et al., 2019; Tiggemann and Slater, 2017; Tseng and Yang, 2015; Twenge and Campbell, 2019; Twenge et al., 2018; van den Eijnden et al., 2018; Wang et al., 2018; Wartberg et al., 2018; Wolke et al., 2017; Woods and Scott, 2016; Yan et al., 2017). Among the included studies, 74 (94%) are quantitative (Aboujaoude et al., 2015; Banjanin et al., 2015; Banyai et al., 2017; Barry et al., 2017; Best et al., 2014; Booker et al., 2018; Bourgeois et al., 2014; Boyle et al., 2016; Brunborg et al., 2017; Colder Carras et al., 2017; Critchlow et al., 2019; Cross et al., 2015; Curtis et al., 2018; de Lenne et al., 2018; de Vries et al., 2016; Erfani and Abedin, 2018; Erreygers et al., 2018; Fahy et al., 2016; Ferguson et al., 2014; Fisher et al., 2016; Foerster and Roosli, 2017; Foody et al., 2017; Fredrick and Demaray, 2018; Frison and Eggermont, 2016, 2017; Geusens and Beullens, 2017, 2018; Hamm et al., 2015; Hanprathet et al., 2015; Harbard et al., 2016; Hase et al., 2015; Houghton et al., 2018; Jafarpour et al., 2017; John et al., 2018; Kim et al., 2019; Kim, 2017; Koo et al., 2015; Lai et al., 2018; Larm et al., 2017, 2019; Marchant et al., 2017; Marengo et al., 2018; Marques et al., 2018; Meier and Gray, 2014; Memon et al., 2018; Merelle et al., 2017; Neira and Barber, 2014; Nesi et al., 2017a, b; Niu et al., 2018; Nursalam et al., 2018; Oberst et al., 2017; O’Connor et al., 2014; Przybylski and Bowes, 2017; Przybylski and Weinstein, 2017; Richards et al., 2015; Rousseau et al., 2017; Salmela-Aro et al., 2017; Sampasa-Kanyinga and Chaput, 2016; Sampasa-Kanyinga and Lewis, 2015; Sampasa-Kanyinga et al., 2018; Scott and Woods, 2018; Settanni et al., 2018; Spears et al., 2015; Tiggemann and Slater, 2017; Tseng and Yang, 2015; Twenge and Campbell, 2019; Twenge et al., 2018; van den Eijnden et al., 2018; Wang et al., 2018; Wartberg et al., 2018; Wolke et al., 2017; Woods and Scott, 2016; Yan et al., 2017), three are qualitative (O’Reilly et al., 2018; Burnette et al., 2017; Throuvala et al., 2019), and two use mixed methods (Best et al., 2015; Holfeld and Mishna, 2019) (see Supplementary Tables 1, 2 in the Supplementary Material for additional details extracted from all included studies). In relation to study design, 45 (57%) used a cross-sectional design (Bourgeois et al., 2014; Ferguson et al., 2014; Meier and Gray, 2014; Neira and Barber, 2014; O’Connor et al., 2014; Banjanin et al., 2015; Hanprathet et al., 2015; Hase et al., 2015; Koo et al., 2015; Sampasa-Kanyinga and Lewis, 2015; Spears et al., 2015; Tseng and Yang, 2015; Frison and Eggermont, 2016; Sampasa-Kanyinga and Chaput, 2016; Woods and Scott, 2016; Banyai et al., 2017; Barry et al., 2017; Brunborg et al., 2017; Colder Carras et al., 2017; Larm et al., 2017, 2019; Merelle et al., 2017; Oberst et al., 2017; Przybylski and Bowes, 2017; Przybylski and Weinstein, 2017; Tiggemann and Slater, 2017; Wolke et al., 2017; Yan et al., 2017; de Lenne et al., 2018; Erreygers et al., 2018; Fredrick and Demaray, 2018; Geusens and Beullens, 2018; Lai et al., 2018; Marengo et al., 2018; Marques et al., 2018; Niu et al., 2018; Nursalam et al., 2018; Sampasa-Kanyinga et al., 2018; Scott and Woods, 2018; Settanni et al., 2018; Wang et al., 2018; Wartberg et al., 2018; Critchlow et al., 2019; Kim et al., 2019; Twenge and Campbell, 2019), 17 used a longitudinal design (Cross et al., 2015; Boyle et al., 2016; de Vries et al., 2016; Fahy et al., 2016; Frison and Eggermont, 2016; Harbard et al., 2016; Foerster and Roosli, 2017; Geusens and Beullens, 2017; Kim, 2017; Nesi et al., 2017a, b; Rousseau et al., 2017; Salmela-Aro et al., 2017; Booker et al., 2018; Houghton et al., 2018; van den Eijnden et al., 2018; Holfeld and Mishna, 2019), seven were systematic reviews (Aboujaoude et al., 2015; Best et al., 2015; Fisher et al., 2016; Marchant et al., 2017; Erfani and Abedin, 2018; John et al., 2018; Memon et al., 2018), two were meta-analyses (Foody et al., 2017: Curtis et al., 2018), one was a causal-comparative study (Jafarpour et al., 2017), one was a review article (Richards et al., 2015), one used a time-lag design (Twenge et al., 2018), one was a scoping review (Hamm et al., 2015), three used a focus-group interview design (Burnette et al., 2017; O’Reilly et al., 2018; Throuvala et al., 2019), and one study used a combined survey and focus-group design (Best et al., 2014).

The most common study settings were schools [*N* = 42 (54%)] (Best et al., 2014; Bourgeois et al., 2014; Meier and Gray, 2014; Neira and Barber, 2014; O’Connor et al., 2014; Banjanin et al., 2015; Hanprathet et al., 2015; Hase et al., 2015; Sampasa-Kanyinga and Lewis, 2015; Frison and Eggermont, 2016; Sampasa-Kanyinga and Chaput, 2016; Woods and Scott, 2016; Banyai et al., 2017; Brunborg et al., 2017; Colder Carras et al., 2017; Foerster and Roosli, 2017; Geusens and Beullens, 2017, 2018; Kim, 2017; Larm et al., 2017, 2019; Merelle et al., 2017; Nesi et al., 2017a, b; Przybylski and Bowes, 2017; Rousseau et al., 2017; Salmela-Aro et al., 2017; Tiggemann and Slater, 2017; de Lenne et al., 2018; Fredrick and Demaray, 2018; Houghton et al., 2018; Lai et al., 2018; Marengo et al., 2018; Niu et al., 2018; Nursalam et al., 2018; Sampasa-Kanyinga et al., 2018; Scott and Woods, 2018; Settanni et al., 2018; van den Eijnden et al., 2018; Wang et al., 2018; Holfeld and Mishna, 2019; Kim et al., 2019). Fourteen of the included studies were based on data from a home setting (Cross et al., 2015; Koo et al., 2015; Spears et al., 2015; Boyle et al., 2016; de Vries et al., 2016; Harbard et al., 2016; Barry et al., 2017; Frison and Eggermont, 2017; Oberst et al., 2017; Yan et al., 2017; Booker et al., 2018; Marques et al., 2018; Wartberg et al., 2018; Critchlow et al., 2019). Eleven publications were reviews or meta-analyses and included primary studies from different settings (Aboujaoude et al., 2015; Best et al., 2015; Hamm et al., 2015; Richards et al., 2015; Fisher et al., 2016; Foody et al., 2017; Marchant et al., 2017; Curtis et al., 2018; Erfani and Abedin, 2018; John et al., 2018; Memon et al., 2018). One study used both a home and school setting (Erreygers et al., 2018), and 11 (14%) of the included studies did not mention the study setting for data collection (Ferguson et al., 2014; Tseng and Yang, 2015; Fahy et al., 2016; Burnette et al., 2017; Jafarpour et al., 2017; Przybylski and Weinstein, 2017; Wolke et al., 2017; O’Reilly et al., 2018; Twenge et al., 2018; Throuvala et al., 2019; Twenge and Campbell, 2019).

### Mental Health Foci of Included Studies

For a visual overview of the mental health foci of the included studies see Figures 2, 3. Most studies had a focus on different negative aspects of mental health, as evident from the frequently used terms in Figures 2, 3. The most studied aspect was depression, with 23 (29%) studies examining the relationship between social media use and depressive symptoms (Ferguson et al., 2014; Neira and Barber, 2014; O’Connor et al., 2014; Banjanin et al., 2015; Richards et al., 2015; Spears et al., 2015; Tseng and Yang, 2015; Fahy et al., 2016; Frison and Eggermont, 2016, 2017; Woods and Scott, 2016; Banyai et al., 2017; Brunborg et al., 2017; Colder Carras et al., 2017; Larm et al., 2017; Nesi et al., 2017a; Salmela-Aro et al., 2017; Fredrick and Demaray, 2018; Houghton et al., 2018; Niu et al., 2018; Twenge et al., 2018; Wang et al., 2018; Wartberg et al., 2018). Twenty of the included studies focused on different aspects of good mental health, such as well-being, happiness, or quality of life (Best et al., 2014, 2015; Bourgeois et al., 2014; Ferguson et al., 2014; Cross et al., 2015; Koo et al., 2015; Richards et al., 2015; Spears et al., 2015; Fahy et al., 2016; Foerster and Roosli, 2017; Przybylski and Bowes, 2017; Przybylski and Weinstein, 2017; Yan et al., 2017; Booker et al., 2018; de Lenne et al., 2018; Erfani and Abedin, 2018; Erreygers et al., 2018; Lai et al., 2018; van den Eijnden et al., 2018; Twenge and Campbell, 2019). Nineteen studies had a more broad-stroke approach, and covered general mental health or psychiatric problems (Aboujaoude et al., 2015; Hanprathet et al., 2015; Hase et al., 2015; Sampasa-Kanyinga and Lewis, 2015; Spears et al., 2015; Fisher et al., 2016; Barry et al., 2017; Jafarpour et al., 2017; Kim, 2017; Merelle et al., 2017; Oberst et al., 2017; Wolke et al., 2017; Marengo et al., 2018; Marques et al., 2018; Sampasa-Kanyinga et al., 2018; Holfeld and Mishna, 2019; Kim et al., 2019; Larm et al., 2019). Eight studies examined the link between social media use and body dissatisfaction and eating disorder symptoms (Ferguson et al., 2014; Meier and Gray, 2014; de Vries et al., 2016; Burnette et al., 2017; Rousseau et al., 2017; Tiggemann and Slater, 2017; Marengo et al., 2018; Wartberg et al., 2018). Anxiety was the focus of seven studies (O’Connor et al., 2014; Koo et al., 2015; Spears et al., 2015; Fahy et al., 2016; Woods and Scott, 2016; Colder Carras et al., 2017; Yan et al., 2017), and 13 studies included a focus on the relationship between alcohol use and social media use (O’Connor et al., 2014; Boyle et al., 2016; Sampasa-Kanyinga and Chaput, 2016; Brunborg et al., 2017; Geusens and Beullens, 2017, 2018; Larm et al., 2017; Merelle et al., 2017; Nesi et al., 2017b; Curtis et al., 2018; Sampasa-Kanyinga et al., 2018; Critchlow et al., 2019; Kim et al., 2019). Seven studies examined the effect of social media use on sleep (Harbard et al., 2016; Woods and Scott, 2016; Yan et al., 2017; Nursalam et al., 2018; Sampasa-Kanyinga et al., 2018; Scott and Woods, 2018; Larm et al., 2019). Five studies saw how drug use and social media use affected each other (O’Connor et al., 2014; Merelle et al., 2017; Sampasa-Kanyinga et al., 2018; Kim et al., 2019; Larm et al., 2019). Self-harm and suicidal behavior was the focus of eleven studies (O’Connor et al., 2014; Sampasa-Kanyinga and Lewis, 2015; Tseng and Yang, 2015; Kim, 2017; Marchant et al., 2017; Merelle et al., 2017; Fredrick and Demaray, 2018; John et al., 2018; Memon et al., 2018; Twenge et al., 2018; Kim et al., 2019). Other areas of focus other than the aforementioned are loneliness, self-esteem, fear of missing out and other non-pathological measures (Neira and Barber, 2014; Banyai et al., 2017; Barry et al., 2017; Colder Carras et al., 2017).

### Social Media Metrics of Included Studies

The studies included in the current scoping review often focus on specific, widely used, social media and social networking services, such as 31 (39%) studies focusing on Facebook (Bourgeois et al., 2014; Meier and Gray, 2014; Banjanin et al., 2015; Cross et al., 2015; Hanprathet et al., 2015; Richards et al., 2015; Sampasa-Kanyinga and Lewis, 2015; Spears et al., 2015; Boyle et al., 2016; de Vries et al., 2016; Frison and Eggermont, 2016; Harbard et al., 2016; Sampasa-Kanyinga and Chaput, 2016; Banyai et al., 2017; Barry et al., 2017; Brunborg et al., 2017; Larm et al., 2017; Merelle et al., 2017; Nesi et al., 2017a, b; Rousseau et al., 2017; Tiggemann and Slater, 2017; Booker et al., 2018; de Lenne et al., 2018; Lai et al., 2018; Marengo et al., 2018; Marques et al., 2018; Memon et al., 2018; Sampasa-Kanyinga et al., 2018; Settanni et al., 2018; Twenge et al., 2018), 11 on Instagram (Sampasa-Kanyinga and Lewis, 2015; Boyle et al., 2016; Sampasa-Kanyinga and Chaput, 2016; Barry et al., 2017; Brunborg et al., 2017; Frison and Eggermont, 2017; Nesi et al., 2017a; Marengo et al., 2018; Memon et al., 2018; Sampasa-Kanyinga et al., 2018), 11 including Twitter (Richards et al., 2015; Sampasa-Kanyinga and Lewis, 2015; Spears et al., 2015; Harbard et al., 2016; Sampasa-Kanyinga and Chaput, 2016; Barry et al., 2017; Brunborg et al., 2017; Merelle et al., 2017; Nesi et al., 2017a; Memon et al., 2018; Sampasa-Kanyinga et al., 2018), and five studies asking about Snapchat (Boyle et al., 2016; Barry et al., 2017; Brunborg et al., 2017; Nesi et al., 2017a; Marengo et al., 2018). Eight studies mentioned Myspace (Richards et al., 2015; Sampasa-Kanyinga and Lewis, 2015; de Vries et al., 2016; Harbard et al., 2016; Sampasa-Kanyinga and Chaput, 2016; Larm et al., 2017; Booker et al., 2018; Sampasa-Kanyinga et al., 2018) and two asked about Tumblr (Barry et al., 2017; Nesi et al., 2017a). Other media such as Skype (Merelle et al., 2017), Youtube (Richards et al., 2015), WhatsApp (Brunborg et al., 2017), Ping (Merelle et al., 2017), Bebo (Booker et al., 2018), Hyves (de Vries et al., 2016), Kik (Brunborg et al., 2017), Ask (Brunborg et al., 2017), and Qzone (Niu et al., 2018) were only included in one study each.

Almost half (*n* = 34, 43%) of the included studies focus on use of social network sites or online communication in general, without specifying particular social media sites, leaving this up to the study participants to decide (Best et al., 2014, 2015; Ferguson et al., 2014; Neira and Barber, 2014; O’Connor et al., 2014; Koo et al., 2015; Tseng and Yang, 2015; Fahy et al., 2016; Woods and Scott, 2016; Burnette et al., 2017; Colder Carras et al., 2017; Foerster and Roosli, 2017; Foody et al., 2017; Geusens and Beullens, 2017, 2018; Jafarpour et al., 2017; Kim, 2017; Marchant et al., 2017; Oberst et al., 2017; Przybylski and Weinstein, 2017; Salmela-Aro et al., 2017; Yan et al., 2017; Curtis et al., 2018; Erfani and Abedin, 2018; Erreygers et al., 2018; Nursalam et al., 2018; Scott and Woods, 2018; van den Eijnden et al., 2018; Wartberg et al., 2018; Critchlow et al., 2019; Holfeld and Mishna, 2019; Larm et al., 2019; Throuvala et al., 2019; Twenge and Campbell, 2019). Seven of the included studies examined the relationship between virtual game worlds or socially oriented video games and mental health (Ferguson et al., 2014; Best et al., 2015; Spears et al., 2015; Yan et al., 2017; van den Eijnden et al., 2018; Larm et al., 2019; Twenge and Campbell, 2019).

In the 79 studies included in this scoping review, several approaches to measuring social media use are utilized. The combination of frequency and duration of social media use is by far the most used measurement of social media use, and 44 (56%) of the included studies collected data on these parameters (Ferguson et al., 2014; Meier and Gray, 2014; Neira and Barber, 2014; Banjanin et al., 2015; Best et al., 2015; Hanprathet et al., 2015; Sampasa-Kanyinga and Lewis, 2015; Tseng and Yang, 2015; Boyle et al., 2016; de Vries et al., 2016; Frison and Eggermont, 2016, 2017; Harbard et al., 2016; Sampasa-Kanyinga and Chaput, 2016; Woods and Scott, 2016; Banyai et al., 2017; Brunborg et al., 2017; Colder Carras et al., 2017; Foerster and Roosli, 2017; Jafarpour et al., 2017; Kim, 2017; Larm et al., 2017, 2019; Merelle et al., 2017; Nesi et al., 2017b; Oberst et al., 2017; Rousseau et al., 2017; Tiggemann and Slater, 2017; Yan et al., 2017; Booker et al., 2018; de Lenne et al., 2018; Erreygers et al., 2018; Houghton et al., 2018; Lai et al., 2018; Marengo et al., 2018; Marques et al., 2018; Niu et al., 2018; Nursalam et al., 2018; Sampasa-Kanyinga et al., 2018; Scott and Woods, 2018; Settanni et al., 2018; Twenge et al., 2018; van den Eijnden et al., 2018; Twenge and Campbell, 2019). Eight studies focused on the relationship between social media addiction or excessive use and mental health (Banjanin et al., 2015; Tseng and Yang, 2015; Banyai et al., 2017; Merelle et al., 2017; Nursalam et al., 2018; Settanni et al., 2018; Wang et al., 2018). Bergen Social Media Addiction Scale is a commonly used questionnaire amongst the included studies (Hanprathet et al., 2015; Banyai et al., 2017; Settanni et al., 2018). Seven studies asked about various specific actions on social media, such as liking or commenting on photos, posting something or participating in a discussion (Meier and Gray, 2014; Koo et al., 2015; Nesi et al., 2017b; Geusens and Beullens, 2018; Marques et al., 2018; van den Eijnden et al., 2018; Critchlow et al., 2019).

Five studies had a specific and sole focus on the link between social media use and alcohol, and examined how various alcohol-related social media use affected alcohol intake (Boyle et al., 2016; Geusens and Beullens, 2017, 2018; Nesi et al., 2017b; Critchlow et al., 2019). Some studies had a more theory-based focus and investigated themes such as peer comparison, social media intrusion or pro-social behavior on social media and its effect on mental health (Bourgeois et al., 2014; Rousseau et al., 2017; de Lenne et al., 2018). One of the included studies looked into night-time specific social media use (Scott and Woods, 2018) and one looked into pre-bedtime social media behavior (Harbard et al., 2016) to study the link between this use and sleep.

Amongst the 79 included studies, only six (8%) studies had participants of one gender (Ferguson et al., 2014; Meier and Gray, 2014; Best et al., 2015; Burnette et al., 2017; Jafarpour et al., 2017; Tiggemann and Slater, 2017). Sixteen studies (20%) did not mention the gender distribution of the participants (Aboujaoude et al., 2015; Best et al., 2015; Hamm et al., 2015; Richards et al., 2015; Fisher et al., 2016; Woods and Scott, 2016; Foody et al., 2017; Marchant et al., 2017; Przybylski and Weinstein, 2017; Curtis et al., 2018; Erfani and Abedin, 2018; John et al., 2018; Memon et al., 2018; O’Reilly et al., 2018; Twenge et al., 2018; Twenge and Campbell, 2019). Several of these were meta-analyses or reviews (Aboujaoude et al., 2015; Best et al., 2014; Curtis et al., 2018; Foody et al., 2017; John et al., 2018; Erfani and Abedin, 2018; Wallaroo, 2020). The studies that included both genders as participants generally had a well-balanced gender distribution with no gender below 40% of the participants. Eight of the studies did not report gender-specific results (Harbard et al., 2016; Nesi et al., 2017b; Curtis et al., 2018; de Lenne et al., 2018; Niu et al., 2018; Nursalam et al., 2018; Wang et al., 2018; Twenge and Campbell, 2019). Of the included studies, gender was seldom examined as an explanatory variable, and other sociodemographic variables (e.g., ethnicity, socioeconomic status) were not included at all.

### Implicit Causation Based on Direction of Association

Sixty-one (77%) of the included studies has social media use as the independent variable and some of the mentioned measurements of mental health as the dependent variable (Aboujaoude et al., 2015; Banjanin et al., 2015; Banyai et al., 2017; Barry et al., 2017; Best et al., 2014; Booker et al., 2018; Bourgeois et al., 2014; Boyle et al., 2016; Brunborg et al., 2017; Colder Carras et al., 2017; Critchlow et al., 2019; Cross et al., 2015; Curtis et al., 2018; de Lenne et al., 2018; de Vries et al., 2016; Erfani and Abedin, 2018; Fahy et al., 2016; Fisher et al., 2016; Foerster and Roosli, 2017; Fredrick and Demaray, 2018; Frison and Eggermont, 2016; Geusens and Beullens, 2018; Hamm et al., 2015; Hanprathet et al., 2015; Harbard et al., 2016; Hase et al., 2015; Holfeld and Mishna, 2019; Jafarpour et al., 2017; John et al., 2018; Kim et al., 2019; Kim, 2017; Lai et al., 2018; Larm et al., 2017, 2019; Marengo et al., 2018; Marques et al., 2018; Meier and Gray, 2014; Memon et al., 2018; Neira and Barber, 2014; Nesi et al., 2017b; Niu et al., 2018; Nursalam et al., 2018; O’Connor et al., 2014; O’Reilly et al., 2018; Przybylski and Bowes, 2017; Przybylski and Weinstein, 2017; Richards et al., 2015; Sampasa-Kanyinga and Chaput, 2016; Sampasa-Kanyinga and Lewis, 2015; Sampasa-Kanyinga et al., 2018; Scott and Woods, 2018; Spears et al., 2015; Tseng and Yang, 2015; Twenge and Campbell, 2019; Twenge et al., 2018; van den Eijnden et al., 2018; Wang et al., 2018; Wartberg et al., 2018; Wolke et al., 2017; Woods and Scott, 2016; Yan et al., 2017). Most of the included studies hypothesize social media use pattern will affect youth mental health in certain ways. The majority of the included studies tend to find a correlation between more frequent social media use and poor well-being and/or mental health (see Supplementary Table 2). The strength of this correlation is however heterogeneous as social media use is measured substantially different across studies. Four (5%) of the included studies focus explicitly on how mental health can affect social media use (Merelle et al., 2017; Nesi et al., 2017a; Erreygers et al., 2018; Settanni et al., 2018). Fourteen studies included a mediating factor or focus on reciprocal relationships between social media use and mental health (Ferguson et al., 2014; Koo et al., 2015; Tseng and Yang, 2015; Frison and Eggermont, 2017; Geusens and Beullens, 2017; Marchant et al., 2017; Oberst et al., 2017; Rousseau et al., 2017; Salmela-Aro et al., 2017; Tiggemann and Slater, 2017; Houghton et al., 2018; Marques et al., 2018; Niu et al., 2018; Wang et al., 2018). An example is a cross-sectional study by Ferguson et al. (2014) suggesting that exposure to social media contribute to later peer competition which was found to be a predictor of negative mental health outcomes such as eating disorder symptoms.

### Cyberbullying as a Nexus

Thirteen of the 79 (17%) included studies investigated cyberbullying as the measurement of social media use (Aboujaoude et al., 2015; Cross et al., 2015; Hamm et al., 2015; Hase et al., 2015; Spears et al., 2015; Fahy et al., 2016; Fisher et al., 2016; Foody et al., 2017; Przybylski and Bowes, 2017; Wolke et al., 2017; Fredrick and Demaray, 2018; John et al., 2018; Holfeld and Mishna, 2019). Most of the systematic reviews and meta-analyses included focused on cyberbullying. A cross-sectional study from 2017 suggests that cyberbullying has similar negative effects as direct or relational bullying, and that cyberbullying is “mainly a new tool to harm victims already bullied by traditional means” (Wolke et al., 2017). A meta-analysis from 2016 concludes that “peer cybervictimization is indeed associated with a variety of internalizing and externalizing problems among adolescents” (Fisher et al., 2016). A systematic review from 2018 concludes that both victims and perpetrators of cyberbullying are at greater risk of suicidal behavior compared with non-victims and non-perpetrators (John et al., 2018).

## Discussion

### Strengths and Limitations of Present Study

The main strength of this scoping review lies in the effort to give a broad overview of published research related to use of social media, and mental health and well-being among adolescents. Although a range of reviews on screen-based activities in general and mental health and well-being exist (Dickson et al., 2018; Orben, 2020), they do not necessarily discern between social media use and other types of technology-based media. Also, some previous reviews tend to be more particular regarding mental health outcome (Best et al., 2014; Seabrook et al., 2016; Orben, 2020), or do not focus on adolescents *per se* (Seabrook et al., 2016). The main limitation is that, despite efforts to make the search strategy as comprehensive and inclusive as possible, we probably have not been able to identify all relevant studies – this is perhaps especially true when studies do include relevant information about social media and mental health/well-being, but this information is part of sub-group analyses or otherwise not the main aim of the studies. In a similar manner, related to qualitative studies, we do not know if our search strategy were as efficient in identifying studies of relevance if this was not the main theme or focus of the study. Despite this, we believe that we were able to strike a balance between specificity and sensitivity in our search strategy.

### Description of Central Themes and Core Concepts

The findings from the present scoping review on social media use and mental health and well-being among adolescents revealed that the majority (about 3/4) of the included studies focused on social media and pathology. The core concepts identified are social media use and its statistical association with symptoms of depression, general psychiatric symptoms and other symptoms of psychopathology. Similar findings were made by Keles et al. (2020) in a systematic review from 2019. Focus on the potential association between social media use and positive outcomes seems to be rarer in the current literature, even though some studies focused on well-being which also includes positive aspects of mental health. Studies focusing on screen-based media in general and well-being is more prevalent than studies linking social media specifically with well-being (Orben, 2020). The notion that excessive social media use is associated with poor mental health is well established within mainstream media. Our observation that this preconception seems to be the starting point for much research is not conducive to increased knowledge, but also alluded to elsewhere (Coyne et al., 2020).

### Why the Focus on Poor Mental Health/Pathology?

The relationship between social media and mental health is likely to be complex, and social media use can be beneficial for maintaining friendships and enriching social life (Seabrook et al., 2016; Birkjær and Kaats, 2019; Coyne et al., 2020; Orben, 2020). This scoping review reveals that the majority of studies focusing on effects of social media use has a clearly stated focus on pathology and detrimental results of social media use. Mainstream media and the public discourse has contributed in creating a culture of fear around social media, with a focus on its negative elements (Ahn, 2012; O’Reilly et al., 2018). It is difficult to pin-point why the one-sided focus on the negative effects of social media has been established within the research literature. But likely reasons are elements of “moral panic,” and reports of increases in mental health problems among adolescents in the same period that social media were introduced and became wide-spread (Birkjær and Kaats, 2019). The phenomenon of moral panic typically resurges with the introduction and increasing use of new technologies, as happened with video games, TV, and radio (Mueller, 2019).

### The Metrics of Social Media

Social media trends change rapidly, and it is challenging for the research field to keep up. The included studies covered some of the most frequently used social media, but the amount of studies focusing on each social media did not accurately reflect the contemporary distribution of users. Even though sites such as Instagram and Snapchat were covered in some studies, the coverage did not do justice to the amount of users these sites had. Newer social media sites such as TikTok were not mentioned in the included studies even though it has several hundred million daily users (Mediakix, 2019; Wallaroo, 2020).

Across the included studies there was some variation in how social media were gauged, but the majority of studies focused on the mere frequency and duration of use. There were little focus on separating between different forms of (inter)actions on social media, as these can vary between being a victim of cyberbullying to participating in healthy community work. Also, few studies differentiated between types of actions (i.e., posting, scrolling, reading), active and passive modes of social media use (i.e., production versus consumption, and level of interactivity), a finding similar to other reports (Seabrook et al., 2016; Verduyn et al., 2017; Orben, 2020). There is reason to believe that different modes of use on social media platforms are differentially associated with mental health, and a recent narrative review highlight the need to address this in future research (Orben, 2020). One of the included studies found for instance that it is not the total time spent on Facebook or the internet, but the specific amount of time allocated to photo-related activities that is associated with greater symptoms of eating disorders such as thin ideal internalization, self-objectification, weight dissatisfaction, and drive for thinness (Meier and Gray, 2014). This observation can possibly be explained by social comparison mechanisms (Appel et al., 2016) and passive use of social media (Verduyn et al., 2017). The lack of research differentiating social media use and its association with mental health is an important finding of this scoping review and will hopefully contribute to this being included in future studies.

Few studies examined the motivation behind choosing to use social media, or the mental health status of the users when beginning a social media session. It has been reported that young people sometimes choose to enter sites such as Facebook and Twitter as an escape from threats to their mental health such as experiencing overwhelming pressure in daily life (Boyd, 2014). This kind of escapism can be explained through uses and gratifications theory [see for instance (Coyne et al., 2020)]. On the other hand, more recent research suggest that additional motivational factors may include the need to control relationships, content, presentation, and impressions (Throuvala et al., 2019), and it is possible that social media use can act as an reinforcement of adolescents’ current moods and motivations (Birkjær and Kaats, 2019). Regardless, it seems obvious that the interplay between online and offline use and underlying motivational mechanisms needs to be better understood.

There has also been some questions about the accuracy when it comes to deciding the amount and frequency of one’s personal social media use. Without measuring duration and frequency of use directly and objectively it is unlikely that subjective self-report of general use is reliable (Kobayashi and Boase, 2012; Scharkow, 2016, 2019; Naab et al., 2019). Especially since the potential for social media use is almost omnipresent and the use itself is diverse in nature. Also, due to processes such as social desirability, it is likely that some participants report lower amounts of social media use as excessive use is seen largely undesirable (Krumpal, 2013). Inaccurate reporting of prior social media use could also be a threat to the validity of the reported numbers and thus bias the results reported. Real-time tracking of actual use and modes of use is therefore recommended in future studies to ensure higher accuracy of these aspects of social media use (Coyne et al., 2020; Orben, 2020), despite obvious legal and ethical challenges. Another aspect of social media use which does not seem to be addressed is potential spill-over effects, where use of social media leads to potential interest in or thinking about use of – and events or contents on – social media when the individual is offline. When this aspect has been addressed, it seems to be in relation to preoccupations and with a focus on excessive use or addictive behaviors (Griffiths et al., 2014). Conversely, given the ubiquitous and important role of social media, experiences on social media – for better or for worse – are likely to be interconnected with the rest of an individual’s lived experience (Birkjær and Kaats, 2019).

### The Studies Seem to Implicitly Think That the Use of Social Media “Causes”/“Affects” Mental Health (Problems)

Most of the included studies establish an implicit causation between social media and mental health. It is assumed that social media use has an impact on mental health. The majority of studies included establish some correlation between more frequent use of social media and poor well-being/mental health, as evident from Supplementary Table 2. As formerly mentioned, most of the included studies are cross-sectional and cannot shed light into temporality or cause-and-effect. In total, only 16 studies had a longitudinal design, using different types of regression models, latent growth curve models and cross-lagged models. Yet there seems to be an unspoken expectation that the direction of the association is social media use affecting mental health. The reason for this supposition is unclear, but again it is likely that the mainstream media discourse dominated by mostly negative stories and reports of social media use has some impact together with the observed moral panic.

With the increased popularity of social media and internet arrived a reduction of face-to-face contact and supposed increased social isolation (Kraut et al., 1998; Espinoza and Juvonen, 2011). This view is described as the displacement hypothesis [see for instance (Coyne et al., 2020)]. Having a thriving social life and community with meaningful relations are for many considered vital for well-being and good mental health, and the supposed reduction of sociality were undoubtedly met with skepticism by some. Social media use has increased rapidly among young people over the last two decades along with reports that mental health problems are increasing. Several studies report that there is a rising prevalence of symptom of anxiety and depression among our adolescents (Bor et al., 2014; Olfson et al., 2015). The observation that increases in social media use and mental health issues happened in more or less the same time period can have contributed to focus on how use of social media affects mental health problems.

The existence of an implicit causation is supported by the study variables chosen and the lack of positively worded outcomes. Depression, anxiety, alcohol use, psychiatric problems, suicidal behavior and eating disorders are amongst the most studied outcome-variables. On the other side of the spectrum we have well-being, which can oscillate from positive to negative, whilst the measures of pathology only vary from “ill” to “not ill” with positive outcomes not possible.

### What Is the Gap in the Literature?

The current literature on social media and mental health among youth is still developing and has several gaps and shortcomings, as evident from this scoping review and other publications (Seabrook et al., 2016; Coyne et al., 2020; Keles et al., 2020; Orben, 2020). Some of the gaps and shortcomings in the field we propose solutions for has been identified in a systematic review from 2019 by Keles et al. (2020). The majority of the included studies in the current scoping review were cross-sectional, were limited in their inclusion of potential confounders and 3rd variables such as sociodemographics and personality, preventing knowledge about possible cause-and-effect between social media and mental health. There is a lack of longitudinal studies examining the effects of social media over extended periods of time, as well as investigations longitudinally of how mental health impacts social media use. However, since the formal search was ended for this scoping review, some innovative studies have emerged using longitudinal data (Brunborg and Andreas, 2019; Orben et al., 2019; Coyne et al., 2020). More high quality longitudinal studies of social media use and mental health could help us identify the patterns over time and help us learn about possible cause-and-effect relationships, as well as disentangling between- and within-person associations (Coyne et al., 2020; Orben, 2020). Furthermore, both social media use and mental health are complex phenomena in themselves, and future studies need to consider which aspects they want to investigate when trying to understand their relationship. Mechanisms linking social media use and eating disorders are for instance likely to be different than mechanisms linking social media use and symptoms of ADHD.

Our literature search also revealed a paucity of qualitative studies exploring the why’s and how’s of social media use in relation to mental health among adolescents. Few studies examine how youth themselves experience and perceive the relationship between social media and mental health, and the reasons for their continued and frequent use. Qualitatively oriented studies would contribute to a deeper understanding of adolescent’s social media sphere, and their thoughts about the relationship between social media use and mental health [see for instance (Burnette et al., 2017)]. For instance, O’Reilly et al. (2018) found that adolescents viewed social media as a threat to mental well-being, and concluded that they buy into the idea that “inherently social media has negative effects on mental wellbeing” and seem to “reify the moral panic that has become endemic to contemporary discourses.” On the other hand, Weinstein found using both quantitative and qualitative data that adolescents’ perceptions of the relationship between social media use and well-being probably is more nuanced, and mostly positive. Another clear gap in the research literature is the lack of focus on potentially positive aspects of social media use. It is obvious that there are some positive sides of the use of social media, and these also need to be investigated further (Weinstein, 2018; Birkjær and Kaats, 2019). Gender-specific analyses are also lacking in the research literature, and there is reason to believe that social media use have different characteristics between the genders with different relationships to mental health. In fact, recent findings indicate that not only gender should be considered an important factor when investigating the role of social media in adolescents’ lives, but individual characteristics in general (Orben et al., 2019; Orben, 2020). Analyses of socioeconomic status and geographic location are also lacking and it is likely that these factors might play a role the potential association between social media use and mental health. And finally, several studies point to the fact that social media potentially could be a fruitful arena for promoting mental well-being among youth, and developing mental health literacy to better equip our adolescents for the challenges that will surely arise (O’Reilly et al., 2018; Teesson et al., 2020).

## Conclusion

Research into the association between social media use and mental health and well-being among adolescents is rapidly emerging. The field is characterized by a focus on the association between social media use and negative aspects of mental health and well-being, and where studies focusing on the potentially positive aspects of social media use are lacking. Presently, the majority of studies in the field are quantitatively oriented, with most utilizing a cross-sectional design. An increase in qualitatively oriented studies would add to the field of research by increasing the understanding of adolescents’ social-media life and their own experiences of its association with mental health and well-being. More studies using a longitudinal design would contribute to examining the effects of social media over extended periods of time and help us learn about possible cause-and-effect relationships. Few studies look into individual factors, which may be important for our understanding of the association. Social media use and mental health and well-being are complex phenomena, and future studies could benefit from specifying the type of social media use they focus on when trying to understand its link to mental health. In conclusion, studies including more specific aspects of social media, individual differences and potential intermediate variables, and more studies using a longitudinal design are needed as the research field matures.

## Author Contributions

JS conceptualized the review approach and provided general guidance to the research team. VS and JS drafted the first version of this manuscript. JS, GH, and LA developed the draft further based on feedback from the author group. All authors reviewed and approved the final version of the manuscript and have made substantive intellectual contributions to the development of this manuscript.

## Conflict of Interest

The authors declare that the research was conducted in the absence of any commercial or financial relationships that could be construed as a potential conflict of interest.

## Footnotes

1 https://rayyan.qcri.org/welcome
