# Supplementary material for: Social Media Use and Mental Health and Well-Being Among Adolescents – A Scoping Review
Source: Front Psychol. 2020 Aug 14;11:1949. doi: 10.3389/fpsyg.2020.01949 (PMC7457037; doi:10.3389/fpsyg.2020.01949)
Supplement: Supplementary file 1 [file Table_1.DOCX]

Table 1: Overview of study characteristics of included studies, and frequently employed mental health indicators and social media measures.

| **Study characteristics** | **Description (N and % of total number of studies)** | | | | |
| --- | --- | --- | --- | --- | --- |
| Study type | 74 (94%) Quantitative | 3 (4%) Qualitative | 2 (<3%) Mixed-methods |  |  |
| Study design | 45 (57%) Cross-sectional | 17 (22%) Longitudinal | 7 (9%) Systematic reviews | 2 (<3%) Systematic reviews and meta-analysis | 8 (10%) Other |
| Study setting | 42 (53%) School | 14 (18%) Home setting | 11 (14%) Reviews or meta-nalysis | 11 (14%) Did not specify study setting | 1 (<1%) Other |
| **Mental health indicators** |  |  |  |  |  |
| Main measure^a^ | 23 (29%) Depression | 20 (25%) Aspects of good mental health | 18 (23%) General mental health or psychiatric problems | 8 (10%) Body dissatisfaction or symptoms of eating disorder | 13 (16%) Alcohol use |
| **Measures of social media** |  |  |  |  |  |
|  |  |  |  |  |  |
| Specific platform-focus^a^ | 31 (39%) Facebook | 11 (14%) Instagram | 11 (14%) Twitter | 5 (6%) Snapchat |  |
| Type of use^a^ | 44 (44%) Frequency and duration of use | 7 (9%) Excessive use | 7 (9%) Focused on specific actions (posting, liking etc) |  |  |
|  |  |  |  |  |  |

^a^Categories not mutually exclusive as one study could focus on more than one aspect.
