# Supplementary material for: Social Media Use and Mental Health and Well-Being Among Adolescents – A Scoping Review
Source: Front Psychol. 2020 Aug 14;11:1949. doi: 10.3389/fpsyg.2020.01949 (PMC7457037; doi:10.3389/fpsyg.2020.01949)
Supplement: Supplementary file 2 [file Table_2.DOCX]

Appendix 1: Overview of included studies.

**Contents**

[Study # 1: «Relationship between internet use and depression: Focus on physiological mood oscillations, social networking and online addictive behavior» 5](#_Toc34659542)

[Study # 2: «Problematic Social Media Use: Results from a Large-Scale Nationally Representative Adolescent Sample» 6](#_Toc34659543)

[Study # 3: «Adolescent social media use and mental health from adolescent and parent perspectives» 7](#_Toc34659544)

[Study # 4: «I've 500 friends, but who are my mates? Investigating the influence of online friend networks on adolescent wellbeing» 8](#_Toc34659545)

[Study # 5: «Different digital paths to the keg? How exposure to peers' alcohol-related social media content influences drinking among male and female first-year college students» 9](#_Toc34659546)

[Study # 6: «Social media use and episodic heavy drinking among adolescents» 10](#_Toc34659547)

[Study # 7: «Video gaming in a hyperconnected world: A cross-sectional study of heavy gaming, problematic gaming symptoms, and online socializing in adolescents» 11](#_Toc34659548)

[Study # 8: «Meta-analysis of the association of alcohol-related social media use with alcohol consumption and alcohol-related problems in adolescents and young adults» 12](#_Toc34659549)

[Study # 9: «Picture-perfect lives on social media: A cross-national study on the role of media ideals in adolescent well-being» 13](#_Toc34659550)

[Study # 10: «Adolescents' depressive symptoms and subsequent technology-based interpersonal behaviors: A multi-wave study» 14](#_Toc34659551)

[Study # 11: «Adolescent self-harm: a school-based study in Northern Ireland» 15](#_Toc34659552)

[Study # 12: « Social networking and the social and emotional wellbeing of adolescents in Australia» 16](#_Toc34659553)

[Study # 13: «Feel good, do good online? Spillover and crossover effects of happiness on adolescents' online prosocial behavior» 17](#_Toc34659554)

[Study # 14: «A latent class analysis on adolescents media use and associations with health related quality of life» 18](#_Toc34659555)

[Study # 15: «Exploring the relationships between different types of Facebook use, perceived online social support, and adolescents' depressed mood» 19](#_Toc34659556)

[Study # 16: «Browsing, posting, and liking on instagram: The reciprocal relationships between different types of instagram use and adolescents' depressed mood» 20](#_Toc34659557)

[Study # 17: «What's Keeping Teenagers Up? Prebedtime Behaviors and Actigraphy-Assessed Sleep Over School and Vacation» 21](#_Toc34659558)

[Study # 18: «Reciprocal relationships between trajectories of depressive symptoms and screen media use during adolescence» 22](#_Toc34659559)

[Study # 19: «The impact of online social networking on adolescent psychological well-being (WB): A population-level analysis of Korean school-aged children» 23](#_Toc34659560)

[Study # 20: «The increased trend of non-drinking alcohol among adolescents: what role do internet activities have? » 24](#_Toc34659561)

[Study # 21: «A systematic review of the relationship between internet use, self-harm and suicidal behaviour in young people: The good, the bad and the unknown» 25](#_Toc34659562)

[Study # 22: «Highly-visual social media and internalizing symptoms in adolescence: The mediating role of body image concerns» 26](#_Toc34659563)

[Study # 23: «Facebook photo activity associated with body image disturbance in adolescent girls» 27](#_Toc34659564)

[Study # 24: «The role of online social networking on deliberate self-harm and suicidality in adolescents: A systematized review of literature» 28](#_Toc34659565)

[Study # 25: «Which health-related problems are associated with problematic video-gaming or social media use in adolescents? A large-scale cross-sectional study» 29](#_Toc34659566)

[Study # 26: «Social networking site use: Linked to adolescents' social self-concept, self-esteem, and depressed mood» 30](#_Toc34659567)

[Study # 27: «Friends' alcohol-related social networking site activity predicts escalations in adolescent drinking: Mediation by peer norms» 31](#_Toc34659568)

[Study # 28: «Association between insomnia and social network site use in Indonesian adolescents» 32](#_Toc34659569)

[Study # 29: «Negative consequences from heavy social networking in adolescents: The mediating role of fear of missing out» 33](#_Toc34659570)

[Study # 30: «Is social media bad for mental health and wellbeing? Exploring the perspectives of adolescents» 34](#_Toc34659571)

[Study # 31: «A large-scale test of the goldilocks hypothesis» 35](#_Toc34659572)

[Study # 32: «Use of social networking sites and alcohol consumption among adolescents» 36](#_Toc34659573)

[Study # 33: «Use of social media is associated with short sleep duration in a dose-response manner in students aged 11 to 20 years» 37](#_Toc34659574)

[Study # 34: «Frequent use of social networking sites is associated with poor psychological functioning among children and adolescents» 38](#_Toc34659575)

[Study # 35: «Fear of missing out and sleep: Cognitive behavioural factors in adolescents' nighttime social media use» 39](#_Toc34659576)

[Study # 36: «The interplay between ADHD symptoms and time perspective in addictive social media use: A study on adolescent Facebook users» 40](#_Toc34659577)

[Study # 37: «Media use is linked to lower psychological well-being: Evidence from three datasets» 41](#_Toc34659578)

[Study # 38: «The impact of heavy and disordered use of games and social media on adolescents' psychological, social, and school functioning» 42](#_Toc34659579)

[Study # 39: «Social networking sites addiction and adolescent depression: A moderated mediation model of rumination and self-esteem» 43](#_Toc34659580)

[Study # 40: «#Sleepyteens: Social media use in adolescence is associated with poor sleep quality, anxiety, depression and low self-esteem» 44](#_Toc34659581)

[Study # 41: «Online communication, social media and adolescent wellbeing: A systematic narrative review» 45](#_Toc34659582)

[Study # 42: «Gender differences in the associations between age trends of social media interaction and well-being among 10-15 year olds in the UK» 46](#_Toc34659583)

[Study # 43: «Adolescents' social network site use, peer appearance-related feedback, and body dissatisfaction: Testing a mediation model» 47](#_Toc34659584)

[Study # 44: «Concurrent and prospective analyses of peer, television and social media influences on body dissatisfaction, eating disorder symptoms and life satisfaction in adolescent girls» 48](#_Toc34659585)

[Study # 45: «Bullying and cyberbullying studies in the school-aged population on the island of Ireland: A meta-analysis» 49](#_Toc34659586)

[Study # 46: «The reciprocal associations between sharing alcohol references on social networking sites and binge drinking: A longitudinal study among late adolescents» 50](#_Toc34659587)

[Study # 47: «Comparing personality traits, mental health and self-esteem in users and non-users of social networks» 51](#_Toc34659588)

[Study # 48: «The double meaning of online social space: Three-way interactions among social anxiety, online social behavior, and offline social behavior» 52](#_Toc34659589)

[Study # 49: «The role of online social network chatting for alcohol use in adolescence: Testing three peer-related pathways in a Swedish population-based sample» 53](#_Toc34659590)

[Study # 50: «Qzone use and depression among Chinese adolescents: A moderated mediation model» 54](#_Toc34659591)

[Study # 51: «Impact of social media on the health of children and young people» 55](#_Toc34659592)

[Study # 52: «The reciprocal and indirect relationships between passive Facebook use, comparison on Facebook, and adolescents' body dissatisfaction» 56](#_Toc34659593)

[Study # 53: «The dark side of internet use: Two longitudinal studies of excessive internet use, depressive symptoms, school burnout and engagement among Finnish early and late adolescents» 57](#_Toc34659594)

[Study # 54: «Facebook and body image concern in adolescent girls: A prospective study» 58](#_Toc34659595)

[Study # 55: «Internet use and web communication networks, sources of social support, and forms of suicidal and nonsuicidal self-injury among adolescents: Different patterns between genders» 59](#_Toc34659596)

[Study # 56: «Increases in depressive symptoms, suicide-related outcomes, and suicide rates among U.S. adolescents after 2010 and links to increased new media screen time» 60](#_Toc34659597)

[Study # 57: «Associations among screen time and unhealthy behaviors, academic performance, and well-being in Chinese adolescents» 61](#_Toc34659598)

[Study # 58: «Cyberbullying: Review of an old problem gone viral» 62](#_Toc34659599)

[Study # 59: «A longitudinal study of the social and emotional predictors and consequences of cyber and traditional bullying victimisation» 63](#_Toc34659600)

[Study # 60: «Longitudinal associations between cyberbullying involvement and adolescent mental health» 64](#_Toc34659601)

[Study # 61: «Peer cybervictimization among adolescents and the associated internalizing and externalizing problems: A meta-analysis» 65](#_Toc34659602)

[Study # 62: «Peer victimization and suicidal ideation: The role of gender and depression in a school-based sample» 66](#_Toc34659603)

[Study # 63: «Prevalence and effect of cyberbullying on children and young people: A scoping review of social media studies» 67](#_Toc34659604)

[Study # 64: «Impacts of traditional bullying and cyberbullying on the mental health of middle school and high school students» 68](#_Toc34659605)

[Study # 65: «Self-Harm, suicidal behaviours, and cyberbullying in children and young people: Systematic review» 69](#_Toc34659606)

[Study # 66: «Cyberbullying and adolescent well-being in England: a population-based cross-sectional study» 70](#_Toc34659607)

[Study # 67: «Cyberbullying, help-seeking and mental health in young Australians: implications for public health» 71](#_Toc34659608)

[Study # 68: «Cyberbullying: a storm in a teacup? » 72](#_Toc34659609)

[Study # 69: «Understanding adolescent students' use of facebook and their subjective wellbeing: A gender-based comparison» 73](#_Toc34659610)

[Study # 70: «Depressive symptoms in adolescents» 74](#_Toc34659611)

[Study # 71: «Facebook addiction and its relationship with mental health among Thai high school students» 75](#_Toc34659612)

[Study # 72: «Facebook: Risks and opportunities in Brazilian and Portuguese youths with different levels of psychosocial adjustment» 76](#_Toc34659613)

[Study # 73: «The association between social networking sites and alcohol abuse among Belgian adolescents: The role of attitudes and social norms» 77](#_Toc34659614)

[Study # 74: «Sex differences in the association between cyberbullying victimization and mental health, substance use, and suicidal ideation in adolescents» 78](#_Toc34659615)

[Study # 75: «Participation with alcohol marketing and user-created promotion on social media, and the association with higher-risk alcohol consumption and brand identification among adolescents in the UK» 79](#_Toc34659616)

[Study # 76: «Motivational processes and dysfunctional mechanisms of social media use among adolescents: A qualitative focus group study» 80](#_Toc34659617)

[Study # 77: «Internalizing symptoms and externalizing problems: Risk factors for or consequences of cyber victimization? » 81](#_Toc34659618)

[Study # 78: «Impacts of the use of social network sites on users' psychological well‐being: A systematic review» 82](#_Toc34659619)

[Study # 79: «“I don’t need people to tell me I’m pretty on social media:” A qualitative study of social media and body image in early adolescent girls» 83](#_Toc34659620)

# Study # 1: **«**Relationship between internet use and depression: Focus on physiological mood oscillations, social networking and online addictive behavior**»**

| **Title** | Relationship between internet use and depression: Focus on physiological mood oscillations, social networking and online addictive behavior |
| --- | --- |
| **Authors** | Banjanin, N., Banjanin, N., Dimitrijevic, I., Pantic, I. |
| **Year** | 2015 |
| **Journal name** | Computers in Human Behavior |
| **Aims** | Investigate the potential relationship between internet addiction and depression in adolescents. |
| **Study design** | Cross-sectional |
| **Study setting** | School setting |
| **Participants** | 336 |
| **Gender distribution** | 116 males |
| **Type of social media use** | The average time spent on social networking, number of friends on their Facebook accounts as well as the number of self-portrait photographs posted on their Facebook account (if any). |
| **How social media was assessed** | Young Internet Addiction Test (IAT) as well as general questions related to internet and social networking site (SNS) use. |
| **Mental Health or well-being measure** | Depression |
| **Type of scales used** | Center for Epidemiologic Studies of Depression Scale for Children (CES-DC), IAT and general questions related to internet and SNS use. |
| **Report gender differences** | Yes |
| **Main findings** | The results of our study indicate that internet use and level of internet addiction measured with IAT scale are positively correlated with depressive symptoms |

# Study # 2: **«**Problematic Social Media Use: Results from a Large-Scale Nationally Representative Adolescent Sample**»**

| **Title** | Problematic social media use: Results from a large-scale nationally representative adolescent sample |
| --- | --- |
| **Authors** | Banyai, F., Zsila, A., Kiraly, O., Maraz, A., Elekes, Z., Griffiths, M. D., Andreassen, C. S., Demetrovics, Z. |
| **Year** | 2017 |
| **Journal name** | PLoS ONE [Electronic Resource] |
| **Aims** | 1. To test the psychometric properties of the Bergen Social Media Addiction Scale (BSMAS) using a nationally representative (Hungarian) adolescent sample. 2. To assess the prevalence of problematic social media use in a nationally representative adolescent sample. |
| **Study design** | Cross-sectional |
| **Study setting** | School setting |
| **Participants** | 6664 |
| **Gender distribution** | 51 % males |
| **Type of social media use** | Facebook |
| **How social media was assessed** | Weekly social media use and Bergen Social Media Addiction Scale |
| **Mental Health or well-being measure** | Self-esteem, depression |
| **Type of scales used** | Rosenberg’s Self-Esteem Scale, Center of Epidemiological Studies Depression-Scale. |
| **Report gender differences** | Yes |
| **Main findings** | In conclusion, the results of the present study suggest that the Bergen Social Media Addiction Scale is a psychometrically valid scale that is an appropriate tool to identify the signs of risky social media use among adolescents. This instrument may be especially useful in school environments to identify those adolescents who are at-risk of problematic social media use and therefore could be utilized in prevention and intervention programs (i.e., content-control software, counseling, cognitive-behavioral therapy). |

# Study # 3: **«**Adolescent social media use and mental health from adolescent and parent perspectives**»**

| **Title** | Adolescent social media use and mental health from adolescent and parent perspectives |
| --- | --- |
| **Authors** | Barry, C. T., Sidoti, C. L., Briggs, S. M., Reiter, S. R., Lindsey, R. A. |
| **Year** | 2017 |
| **Journal name** | Journal of Adolescence |
| **Aims** | To investigate adolescent and parent reports of adolescent social media use and its relation to adolescent psychosocial adjustment. |
| **Study design** | Cross-sectional |
| **Study setting** | Home setting |
| **Participants** | 226 |
| **Gender distribution** | 55 males, 7 unreported |
| **Type of social media use** | Adolescents were first asked which social media applications, if any, they use (i.e.,“Facebook,”“Twitter,”“Snapchat,”“Tumblr,”“Instagram,”“Other”). |
| **How social media was assessed** | Adolescents completed a survey, developed for this study, of their social media use with content of items directly reflecting the parameters of interest. |
| **Mental Health or well-being measure** | DSM checklist, fear of missing out (FoMo), loneliness |
| **Type of scales used** | DSM checklist, Fear of missing out survey, UCLA loneliness scale, self-developed social media checklist |
| **Report gender differences** | Yes |
| **Main findings** | Parent and adolescent reports of the number of adolescents’ social media accounts were moderately correlated with parent-reported DSM-5 symptoms of inattention, hyperactivity/impulsivity, oppositional defiant disorder, anxiety, and depressive symptoms, as well as adolescent-reported FoMO and loneliness. Lastly, anxiety and depressive symptoms were highest among adolescents with a relatively high number of parent-reported social media accounts and relatively high FoMO. |

# Study # 4: **«**I've 500 friends, but who are my mates? Investigating the influence of online friend networks on adolescent wellbeing**»**

| **Title** | I've 500 friends, but who are my mates? Investigating the influence of online friend networks on adolescent wellbeing |
| --- | --- |
| **Authors** | Best, P., Taylor, B., Manktelow, R. |
| **Year** | 2015 |
| **Journal name** | Journal of Public Mental Health |
| **Aims** | The purpose of this paper is to investigate the relationship between online friend networks and the mental well-being (MWB) of adolescent males. |
| **Study design** | Survey & focus group interviews |
| **Study setting** | School setting |
| **Participants** | 521 + 56 |
| **Gender distribution** | 100 % males |
| **Type of social media use** | Chatting with friends, posting comments, uploading pictures, playing games, chatting with strangers |
| **How social media was assessed** | Online social networking (OSN) habits and friendship survey which examined OSN and internet use. |
| **Mental Health or well-being measure** | Well-being |
| **Type of scales used** | Warwick-Edinburgh Mental Wellbeing Scale, OSN habits and friendship survey |
| **Report gender differences** | Not relevant |
| **Main findings** | A positive relationship (p<0.05) was found between the number of online friends and well-being scores. However, higher numbers of online friends were also associated with increases in negative online experiences namely, receiving embarrassing posts online or risky activities such as, chatting frequently with strangers. |

# Study # 5: **«**Different digital paths to the keg? How exposure to peers' alcohol-related social media content influences drinking among male and female first-year college students**»**

| **Title** | Different digital paths to the keg? How exposure to peers' alcohol-related social media content influences drinking among male and female first-year college students |
| --- | --- |
| **Authors** | Boyle, S. C., LaBrie, J. W., Froidevaux, N. M., Witkovic, Y. D. |
| **Year** | 2016 |
| **Journal name** | Addictive Behaviors |
| **Aims** | The current research aims to advance the understanding of alcohol-related social media sites influence among college students by addressing these re-maining questions. |
| **Study design** | Longitudinal |
| **Study setting** | Home setting |
| **Participants** | 412 |
| **Gender distribution** | 36% males |
| **Type of social media use** | Facebook, Instagram, Snapchat |
| **How social media was assessed** | Frequency of checking Facebook, Instagram, and Snapchat, Frequency of Seeing Alcohol-Related Content on Facebook, Instagram,and Snapchat, Exposure to Peers' Alcohol-Related Social Media Site Content |
| **Mental Health or well-being measure** | Alcohol use |
| **Type of scales used** |  |
| **Report gender differences** | Yes |
| **Main findings** | Consistent with previous research documenting college students' frequently recording their risky drinking behaviors on social media sites, first-year students in this study reported observing alcohol-related content posted by peers on Facebook, Instagram, and Snapchat. Exposure to this content during the first 6 weeks of college predicted their own alcohol consumption 6 months later, even after controlling for students' and close friends’ initial drinking. Male students evidenced a much stronger predictive relationship between first semester social media site alcohol exposure and second semester drinking than did females. Further, perceptions of descriptive drinking norms, college alcohol beliefs and enhancement drinking motives, only partially explained the stronger relationship among male students. |

# Study # 6: **«**Social media use and episodic heavy drinking among adolescents**»**

| **Title** | Social media use and episodic heavy drinking among adolescents |
| --- | --- |
| **Authors** | Brunborg, G. S., Andreas, J. B., Kvaavik, E. |
| **Year** | 2017 |
| **Journal name** | Psychological Reports |
| **Aims** | The current study estimated the association between the amount of time adolescents spend on social media and the risk of episodic heavy drinking |
| **Study design** | Cross-sectional |
| **Study setting** | School setting |
| **Participants** | 851 |
| **Gender distribution** | 46 % males |
| **Type of social media use** | Facebook, Snapchat, WhatsApp, Twitter, Instagram, Kik, Ask |
| **How social media was assessed** | Frequency and quantity of social media use |
| **Mental Health or well-being measure** | Episodic heavy drinking, depression |
| **Type of scales used** | 9-item Severity Measure for Depression, self-developed measures |
| **Report gender differences** | Yes |
| **Main findings** | The results from the current study indicate that more time spent on social media is related to greater likelihood of episodic heavy drinking among adolescents |

# Study # 7: **«**Video gaming in a hyperconnected world: A cross-sectional study of heavy gaming, problematic gaming symptoms, and online socializing in adolescents**»**

| **Title** | Video gaming in a hyperconnected world: A cross-sectional study of heavy gaming, problematic gaming symptoms, and online socializing in adolescents |
| --- | --- |
| **Authors** | Colder Carras, M., Van Rooij, A. J., Van de Mheen, D., Musci, R., Xue, Q. L., Mendelson, T. |
| **Year** | 2017 |
| **Journal name** | Computers in Human Behavior |
| **Aims** | Examining online social interactions along with patterns of video gaming behaviors and game addiction symptoms has the potential to enrich our understanding of disorders related to excessive videogame play |
| **Study design** | Cross-sectional |
| **Study setting** | School setting |
| **Participants** | 9733 |
| **Gender distribution** | 49 % males |
| **Type of social media use** | Video games, social internet use |
| **How social media was assessed** | The Video Game Addiction Test, "Youth were also asked how many days per week and hours per day they used instant messaging, social networking, and three types of games" |
| **Mental Health or well-being measure** | Loneliness, depression, social anxiety, self-esteem, friendship quality |
| **Type of scales used** | Depressive Mood List, UCLA Loneliness Scale, Social Anxiety Scale Revised, Rosenberg's Self-Esteem Scale, Network of Relationships Inventory, |
| **Report gender differences** | Yes |
| **Main findings** | In adolescents, symptoms of video game addiction depend not only on video game play but also on concurrent levels of online communication, and those who are very socially active online report fewer symptoms of game addiction |

# Study # 8: **«**Meta-analysis of the association of alcohol-related social media use with alcohol consumption and alcohol-related problems in adolescents and young adults**»**

| **Title** | Meta-analysis of the association of alcohol-related social media use with alcohol consumption and alcohol-related problems in adolescents and young adults |
| --- | --- |
| **Authors** | Curtis, B. L., Lookatch, S. J., Ramo, D. E., McKay, J. R., Feinn, R. S., Kranzler, H. R. |
| **Year** | 2018 |
| **Journal name** | Alcoholism: Clinical & Experimental Research |
| **Aims** | We examined the relations between young adults’ alcohol-related social media engagement (defined as the posting, liking, commenting, and viewing of alcoholrelated social media content) and their drinking behavior and problems. |
| **Study design** | Meta-analysis |
| **Study setting** | Varied |
| **Participants** | Varied |
| **Gender distribution** | Varied |
| **Type of social media use** | Posting, liking, commenting, and viewing of alcoholrelated social media content |
| **How social media was assessed** | Varied |
| **Mental Health or well-being measure** | Alcohol use |
| **Type of scales used** | Alcohol Use Disorders Identification Test, Timeline Followback Interview, self-developed measures for social media |
| **Report gender differences** | No |
| **Main findings** | We found moderate-sized effects across the 19 studies: greater alcohol-related social media engagement was correlated with both greater self-reported drinking and alcohol-related problems. |

# Study # 9: **«**Picture-perfect lives on social media: A cross-national study on the role of media ideals in adolescent well-being**»**

| **Title** | Picture-perfect lives on social media: A cross-national study on the role of media ideals in adolescent well-being |
| --- | --- |
| **Authors** | de Lenne, O., Vandenbosch, L., Eggermont, S., Karsay, K., Trekels, J. |
| **Year** | 2018 |
| **Journal name** | Media Psychology |
| **Aims** | The current study aims to explore the internalization of professional, social, sexual, and romantic media ideals as an explanatory factor in the relation between social media use and adolescents’ poor mental well-being. |
| **Study design** | Cross-sectional |
| **Study setting** | School setting |
| **Participants** | 1983 |
| **Gender distribution** | 50 % males |
| **Type of social media use** | Facebook and Instagram |
| **How social media was assessed** | Participants reported how much time they spent per day on Facebook or Instagram on a scale ranging from 1 (= never use it to less than 10 minutes) to 7 (= more than 6 hours). |
| **Mental Health or well-being measure** | Mental well-being |
| **Type of scales used** | Self-developed Facebook and Instagram use, Self-developed scale of Internalization of professional, social, sexual, and romantic ideals, The Mental Health Inventory (MHI-5) |
| **Report gender differences** | No |
| **Main findings** | This survey study among 1,983 Austrian, Belgian, Spanish, and South Korean adolescents (aged 12–19, 49.7% girls) addressed this gap and found that Instagram use was positively related to the internalization of professional, social, sexual, and romantic ideals, and Facebook use was positively related to the internalization of social and romantic ideals. In turn, the internalization of sexual ideals was related to poor mental well-being. Furthermore, Facebook use was also directly associated with poor mental well-being. Cross-national differences played an important role in that South Korea significantly differed from Austria, Belgium, and Spain in the relations between social media use, the internalization of social ideals, and poor mental well-being. We also found differences among Belgium, Austria, and Spain in the relations between social media use, the internalization of professional, social, and sexual ideals, and poor mental well-being. |

# Study # 10: **«**Adolescents' depressive symptoms and subsequent technology-based interpersonal behaviors: A multi-wave study**»**

| **Title** | Adolescents' depressive symptoms and subsequent technology-based interpersonal behaviors: A multi-wave study |
| --- | --- |
| **Authors** | Nesi, J., Miller, A. B., Prinstein, M. J. |
| **Year** | 2017 |
| **Journal name** | Journal of Applied Developmental Psychology |
| **Aims** | The current study examined longitudinal relationships between de-pressive symptoms and technology-based social comparison and feed-back-seeking (SCFS) among adolescents over a three-year period, andspecifically examined whether depressive symptoms were predictiveof higher levels of SCFS in the subsequent year |
| **Study design** | Longitudinal |
| **Study setting** | School setting |
| **Participants** | 816 |
| **Gender distribution** | 45 % males |
| **Type of social media use** | Electronic interaction,”defined for participants as“texting, Facebook, and other social media (e.g. Twitter, Instagram, Snapchat,Tumblr”) |
| **How social media was assessed** | The Motivations for Electronic Interaction Scale (MEIS), The Electronic Interaction Scale for Time (EIS_T) |
| **Mental Health or well-being measure** | Depression |
| **Type of scales used** | The Motivations for Electronic Interaction Scale (MEIS), The Electronic Interaction Scale for Time (EIS_T), the Short Mood and Feelings Questionnaire |
| **Report gender differences** | Yes |
| **Main findings** | Results indicated that higher levels of depressive symptoms were concurrently associated with greater SCFS afteraccounting for adolescents' typical patterns of SCFS. For boys only, higher depressive symptoms were prospectively associated with later increases in SCFS. Results highlight the importance of social media as a unique context in which depressed adolescents may be at risk for maladaptive interpersonal behavior |

# Study # 11: **«**Adolescent self-harm: a school-based study in Northern Ireland**»**

| **Title** | Adolescent self-harm: A school-based study in Northern Ireland |
| --- | --- |
| **Authors** | O'Connor, R. C., Rasmussen, S., Hawton, K. |
| **Year** | 2014 |
| **Journal name** | Journal of Affective Disorders |
| **Aims** | This study aimed to determine the prevalence of self-harm in Northern Ireland adolescents and the factors associated with it, including exposure to the Northern Ireland conflict. |
| **Study design** | Cross-sectional |
| **Study setting** | School setting |
| **Participants** | 3596 |
| **Gender distribution** | 1882 males, 3 unknown |
| **Type of social media use** | Social and internet influences |
| **How social media was assessed** | Influence of social media on suicidal behavior |
| **Mental Health or well-being measure** | Smoking, alcohol, drug use, self harm, depression, anxiety |
| **Type of scales used** | The Hospital Anxiety and Depression Scale, Plutchik Impulsivity Scale, self-Concept Scale, the brief version of the Child and Adolescent Perfectionism Scale |
| **Report gender differences** | Yes |
| **Main findings** | The rate of self-harm was lower than elsewhere in the UK/Ireland. The study highlights factors which should be considered in terms of risk assessment. In addition to established risk factors, the findings suggest that more research on the legacy of the Northern Ireland conflict as well as the influence of new technologies warrant urgent attention |

# Study # 12: **«** Social networking and the social and emotional wellbeing of adolescents in Australia**»**

| **Title** | Social networking and the social and emotional wellbeing of adolescents in Australia |
| --- | --- |
| **Authors** | Bourgeois, A., Bower, J., Carroll, A. |
| **Year** | 2014 |
| **Journal name** | Australian Journal of Guidance and Counselling |
| **Aims** | This current study is part of a larger four-phase study investigating how schools promote social connectedness and engagement in their students (See rocedure Section). Part of that data examined how young people connected to others using social networking sites and is the subject of this article. |
| **Study design** | Cross-sectional |
| **Study setting** | School setting |
| **Participants** | 1343 |
| **Gender distribution** | 51% males |
| **Type of social media use** | Online connectedness on Facebook |
| **How social media was assessed** | Self in a Social Context: Virtual Self subscale. The items in these subscales are presented to see if participants indicate that they use social networking websites |
| **Mental Health or well-being measure** | Social and emotional wellbeing |
| **Type of scales used** | The self in a Social Context, Virtual Connectedness Subscale, Strength and Difficulties Questionnaire |
| **Report gender differences** | Yes |
| **Main findings** | These findings suggest that social networking sites, though used differently by males and females, provide an important forum for building social connections across groups. |

# Study # 13: **«**Feel good, do good online? Spillover and crossover effects of happiness on adolescents' online prosocial behavior**»**

| **Title** | Feel good, do good online? Spillover and crossover effects of happiness on adolescents' online prosocial behavior |
| --- | --- |
| **Authors** | Erreygers, S., Vandebosch, H., Vranjes, I., Baillien, E., Witte, H. |
| **Year** | 2018 |
| **Journal name** | Journal of Happiness Studies: An Interdisciplinary Forum on Subjective Well-Being |
| **Aims** | In this study we examine spillover and crossover efects of adolescents’ and their parents’ daily happiness on adolescents’ online prosocial behavior via a daily diary |
| **Study design** | Cross-sectional |
| **Study setting** | School & home setting |
| **Participants** | 136 |
| **Gender distribution** | 67 males |
| **Type of social media use** | Use of social network sites, instant messaging, emailing, texting |
| **How social media was assessed** | Five items assessed adolescents’ online prosocial behavior, and adolescents rated how often they had used digital technologies for interpersonal contact |
| **Mental Health or well-being measure** | Happiness |
| **Type of scales used** | Self-developed scales of happiness and online prosocial behavior, use of digital technologies |
| **Report gender differences** | Yes |
| **Main findings** | The findings suggest that, on a daily level, happiness creates a ripple effect whereby adolescents and parents take their positive emotional states from school and work home, and adolescents act on their happiness by behaving more prosocially online. The strongest spillover and crossover efects were found for girls and their mothers, evoking questions for future research to understand these gender differences. |

# Study # 14: **«**A latent class analysis on adolescents media use and associations with health related quality of life**»**

| **Title** | A latent class analysis on adolescents media use and associations with health related quality of life |
| --- | --- |
| **Authors** | Foerster, M., Roosli, M. |
| **Year** | 2017 |
| **Journal name** | Computers in Human Behavior |
| **Aims** | The aim of this study is to use latent class analysis in a sample 895 Swiss adolescents to classifydifferent media usage types based on eleven media use variables. |
| **Study design** | Longitudinal |
| **Study setting** | School setting |
| **Participants** | 850 |
| **Gender distribution** | 43 % males |
| **Type of social media use** | Quantitative social media use and online gaming |
| **How social media was assessed** | Student's media use was assessed through questionnaire and included detailed questions about their quantitative use of mobile phones and other media devices, as well as questions on different use possibilities like social media use or online gaming. |
| **Mental Health or well-being measure** | Well-being |
| **Type of scales used** | The KIDSCREEN-52 quality of life measure, Mobile Phone Problem Use Scale -10 |
| **Report gender differences** | Yes |
| **Main findings** | Five distinct media use classes could be identified: Low Use, Medium Use, Gaming, Call Preference and High Social Use. |

# Study # 15: **«**Exploring the relationships between different types of Facebook use, perceived online social support, and adolescents' depressed mood**»**

| **Title** | Exploring the relationships between different types of Facebook use, perceived online social support, and adolescents' depressed mood |
| --- | --- |
| **Authors** | Frison, E., Eggermont, S. |
| **Year** | 2016 |
| **Journal name** | Social Science Computer Review |
| **Aims** | The current study aims to provide a deeper understanding of the relationships between different types of Facebook use, perceived online social support, and boys’ and girls’ depressed mood. |
| **Study design** | Cross-sectional |
| **Study setting** | School setting |
| **Participants** | 910 |
| **Gender distribution** | 48 % males |
| **Type of social media use** | Facebook use frequency and type of activity |
| **How social media was assessed** | Active public Facebook use, active private Facebook use and passive Facebook use (being exposed to other users’ Facebook profiles). |
| **Mental Health or well-being measure** | Depression |
| **Type of scales used** | The Center for Epidemiological Studies Depression Scale for Children, self-developed measures |
| **Report gender differences** | Yes |
| **Main findings** | Active and passive Facebook use are differentially related to adolescents’ depressed mood. The results provided support for the hypothesized-mediated impact of perceived online social support, within the relationship between active Facebook use and girls’ depressed mood. Gender plays an important role in the proposed relationships. |

# Study # 16: **«**Browsing, posting, and liking on instagram: The reciprocal relationships between different types of instagram use and adolescents' depressed mood**»**

| **Title** | Browsing, posting, and liking on Instagram: The reciprocal relationships between different types of instagram use and adolescents' depressed mood |
| --- | --- |
| **Authors** | Frison, E., Eggermont, S. |
| **Year** | 2017 |
| **Journal name** | Cyberpsychology, behavior and social networking |
| **Aims** | The present study aims to enhance our understanding of the reciprocal relationships between different types of Instagram use (i.e., browsing, posting, and liking) and adolescents’ depressed mood. |
| **Study design** | Longitudinal |
| **Study setting** | Home setting |
| **Participants** | 671 |
| **Gender distribution** | 39 % males |
| **Type of social media use** | Different types of Instagram use |
| **How social media was assessed** | Using a seven-point Likert scale (1 = never to 7 = several times per day), participants were asked (1) ‘‘How often do you look at photos posted by other Instagram users?’’ (i.e., browsing), (2) ‘‘How often do you post a photo on Instagram?’’ (i.e., posting) |
| **Mental Health or well-being measure** | Depression |
| **Type of scales used** | The Center for Epidemiological Studies Depression Scale for Children (CES-DC) |
| **Report gender differences** | Yes |
| **Main findings** | Overall, we can conclude (1) that adolescents have a higher chance to develop a greater depressed mood when they browse more often through Instagram and (2) that adolescents have a higher chance to post more on Instagram when they have higher levels of depressed mood. |

# Study # 17: **«**What's Keeping Teenagers Up? Prebedtime Behaviors and Actigraphy-Assessed Sleep Over School and Vacation**»**

| **Title** | What's keeping teenagers up? Prebedtime behaviors and actigraphy-assessed sleep over school and vacation |
| --- | --- |
| **Authors** | Harbard, E., Allen, N. B., Trinder, J., Bei, B. |
| **Year** | 2016 |
| **Journal name** | Journal of Adolescent Health |
| **Aims** | The present study aimed to: (1) Characterize patterns of both technology and nontechnology-related pre-bedtime behaviors (PBBs) during school and vacation periods using a longitudinal design. (2) Examine how PBBs relate to objectively measured sleep timing (bedtime and rise time) and duration (total sleep time) controlling for chronotype and sleep-onset-latency (SOL; chronotype was not controlled for in SOL analyses as it shared negligible correlation with SOL). Based on the existing literature, these sleep variables represent key aspects of sleep that might be directly affected by PBBs. (3) Examine whether cognitive presleep arousal is a mechanism mediating significant associations between technology-related PBBs and longer SOL. |
| **Study design** | Longitudinal |
| **Study setting** | Home setting |
| **Participants** | 146 |
| **Gender distribution** | 47 % males |
| **Type of social media use** | Online social networking (e.g.,Facebook, Twitter, Myspace), online chat/discussions(chatroom, instant messengers,including audio and video chats) |
| **How social media was assessed** | The Prebedtime Behavior Questionnaire (PBBQ) is a self-report inventory that assesses frequencies of 25 evening behaviors adolescents commonly engaged in |
| **Mental Health or well-being measure** | Sleep, chronotype, presleep arousal |
| **Type of scales used** | The Prebedtime Behavior Questionnaire, The Morningness-Eveningness Questionnaire, The Presleep Arousal Scale |
| **Report gender differences** | No |
| **Main findings** | Technology-related PBBs video games and online social media were risk factors for shorter and poorer sleep, whereas time with family was protective of sleep duration. |

# Study # 18: **«**Reciprocal relationships between trajectories of depressive symptoms and screen media use during adolescence**»**

| **Title** | Reciprocal relationships between trajectories of depressive symptoms and screen media use during adolescence |
| --- | --- |
| **Authors** | Houghton, S., Lawrence, D., Hunter, S. C., Rosenberg, M., Zadow, C., Wood, L., & Shilton, T. |
| **Year** | 2018 |
| **Journal name** | Journal of Youth and Adolescence |
| **Aims** | The first aim of the current study was to use longitudinal data to identify trajectories of depressive symptoms in adolescents and to consider associations that might exist between trajectory classes and screen use time. The second aim of the study was to use longitudinal data to evaluate whether there are associations between screen use and subsequent depressive symptomatology or between depressive symptomatology and subsequent screen use. |
| **Study design** | Longitudinal |
| **Study setting** | School setting |
| **Participants** | 1749 |
| **Gender distribution** | 53 % males |
| **Type of social media use** | Social networking, gaming |
| **How social media was assessed** | The Screen Based Media Use Scale (SBMUS) (the respondents estimate the time they spent on a typical weekday and a typical weekend day (as used for total screen time) on gaming, social media use etc) |
| **Mental Health or well-being measure** | Depression |
| **Type of scales used** | The Screen Based Media Use Scale (SBMUS), The Children’s Depression Inventory 2 (CDI 2) |
| **Report gender differences** | Yes |
| **Main findings** | No substantial evidence for a longitudinal association between screen use and depressive symptoms was identified, undermining the likelihood that there is a causal link between screen use and subsequent changes in depression, or vice versa. There was however, a temporal association found in those who experienced increases in depressive symptoms over the course of the study. This is a valuable contribution to existing research in that significant increases in screen use time may indicate that a young person’s mental health is deteriorating. |

# Study # 19: **«**The impact of online social networking on adolescent psychological well-being (WB): A population-level analysis of Korean school-aged children**»**

| **Title** | The impact of online social networking on adolescent psychological well-being (WB): A population-level analysis of Korean school-aged children |
| --- | --- |
| **Authors** | Kim, H. H.-s. |
| **Year** | 2017 |
| **Journal name** | International Journal of Adolescence and Youth |
| **Aims** | This study examines the extent to which online media activities are associated with psychological well-being of adolescents. |
| **Study design** | Longitudinal |
| **Study setting** | School setting |
| **Participants** | 2099 |
| **Gender distribution** | 53 % males |
| **Type of social media use** | The frequency of online communication or networking |
| **How social media was assessed** | How often do you (a) chat online or use online messenger services; (b) use email; (c) participate in an online community or club; (d) or use an online bulletin board? Answers coded on a five-point scale (e.g. 5 = very often or 1 = hardly at all) |
| **Mental Health or well-being measure** | Mental health, suicidal thoughts |
| **Type of scales used** | Self-assessment of mental health coded on a five-point scale, with a higher value indicating better health. Self-reported suicidal thought. |
| **Report gender differences** | Yes |
| **Main findings** | The central finding of this study is that there is a strong and negative relationship between online activities (chatting, e-mailing, participating in communities or clubs and using bulletin boards) and self-reported mental health and suicidal ideation among a nationally representative sample of Korean students. |

# Study # 20: **«**The increased trend of non-drinking alcohol among adolescents: what role do internet activities have? **»**

| **Title** | The increased trend of non-drinking alcohol among adolescents: what role do internet activities have? |
| --- | --- |
| **Authors** | Larm, P., Raninen, J., Aslund, C., Svensson, J., Nilsson, K. W. |
| **Year** | 2019 |
| **Journal name** | European Journal of Public Health |
| **Aims** | The aim of the present study is to evaluate a common suggestion in literature, that adolescents do not drink alcohol because they spend more time on the internet, monitored at home, by examining associations between internet activities (social media/chatting and computer gaming) and non-drinking. |
| **Study design** | Cross-sectional |
| **Study setting** | School setting |
| **Participants** | 7089 |
| **Gender distribution** | 50 % males |
| **Type of social media use** | Internet activities included total computer time, time spent on social  media/chatting and time spent using computer games |
| **How social media was assessed** | Internet activities included total computer time, time spent on social  media/chatting and time spent using computer games |
| **Mental Health or well-being measure** | family substance problems, physical abuse, psychosomatic problems, sleeping problems, use of cannabis and use of other illicit drugs, alcohol use |
| **Type of scales used** |  |
| **Report gender differences** | Yes |
| **Main findings** | Internet activities were in general not associated with non-drinking among adolescents aged 15–16 years in Sweden. Although, a weak positive association between computer gaming and non-drinking was found in 2012, this effect benefited the vast majority of the boys. The larger alcohol use among those with extensive social media use/chatting may indicate that these online platforms are arenas where adolescents are exposed for positive alcohol preferences and alcohol advertising without parental supervision. |

# Study # 21: **«**A systematic review of the relationship between internet use, self-harm and suicidal behaviour in young people: The good, the bad and the unknown**»**

| **Title** | A systematic review of the relationship between internet use, self-harm and suicidal behaviour in young people: The good, the bad and the unknown |
| --- | --- |
| **Authors** | Marchant, A., Hawton, K., Stewart, A., Montgomery, P., Singaravelu, V., Lloyd, K., Purdy, N., Daine, K., John, A. |
| **Year** | 2017 |
| **Journal name** | PLoS ONE [Electronic Resource] |
| **Aims** | We aimed to systematically review evidence regarding the potential influence of the internet on self-harm/suicidal behaviour in young people. |
| **Study design** | Systematic review |
| **Study setting** | Varied |
| **Participants** | Varied |
| **Gender distribution** | Varied |
| **Type of social media use** | Varied |
| **How social media was assessed** | Varied |
| **Mental Health or well-being measure** | Self-harm, suicidal behavior |
| **Type of scales used** | Varied |
| **Report gender differences** | Varied |
| **Main findings** | There is significant potential for harm from online behaviour (normalisation, triggering, competition, contagion) but also the potential to exploit its benefits (crisis support, reduction of social isolation, delivery of therapy, outreach). Young people appear to be increasingly using social media to communicate distress, particularly to peers. The focus should now be on how specific mediums’ (social media, video/image sharing) might be used in therapy and recovery. Clinicians working with young people who self-harm or have mental health issues should engage in discussion about internet use. This should be a standard item during assessment. |

# Study # 22: **«**Highly-visual social media and internalizing symptoms in adolescence: The mediating role of body image concerns**»**

| **Title** | Highly-visual social media and internalizing symptoms in adolescence: The mediating role of body image concerns |
| --- | --- |
| **Authors** | Marengo, D., Longobardi, C., Fabris, M., Settanni, M. |
| **Year** | 2018 |
| **Journal name** | Computers in Human Behavior |
| **Aims** | The present study aims at evaluate the association between social media use, and in particular that of HVSM, with body image concerns and internalizing symptoms in a sample of adolescents attending grades 6-11 |
| **Study design** | Cross-sectional |
| **Study setting** | School setting |
| **Participants** | 523 |
| **Gender distribution** | 46 % males |
| **Type of social media use** | Facebook, Instagram, Snapchat etc. |
| **How social media was assessed** | We asked participants to report about daily use (hours per day) of Facebook and highly visual social media (HVSM, i. e, Instagram,Snapchat) |
| **Mental Health or well-being measure** | Body image concerns, internalizing symptoms |
| **Type of scales used** | Body Shape Questionnaire, Strength and Difficulties Questionnaires |
| **Report gender differences** | Yes |
| **Main findings** | Our study highlights the mediating effect of body image concerns on the relationship between use of image- and video-based social media (HVSM) and internalizing symptoms among adolescents. In doing this, our study fills a gap in the literature on the impact use of highly-visual social media is having on adolescents regarding body image and mental-health |

# Study # 23: **«**Facebook photo activity associated with body image disturbance in adolescent girls**»**

| **Title** | Facebook photo activity associated with body image disturbance in adolescent girls |
| --- | --- |
| **Authors** | Meier, E. P., Gray, J. |
| **Year** | 2014 |
| **Journal name** | Cyberpsychology, behavior and social networking |
| **Aims** | The present study examined the relationship between body image and adolescent girls’ activity on the social networking site (SNS) Facebook (FB). |
| **Study design** | Cross-sectional |
| **Study setting** | School setting |
| **Participants** | 103 |
| **Gender distribution** | 0 % males |
| **Type of social media use** | The Facebook questionnaire was created by the authors to assess total Internet and FB use and FB appearance exposure. |
| **How social media was assessed** | The Facebook Questionnaire assessed the frequency of user activity on 24 specific FB features (e.g. create an event, join “grups”). |
| **Mental Health or well-being measure** | Internalization of the thin ideal, appearance comparison, weight satisfaction, drive for thinness, self-objectification |
| **Type of scales used** | The 5-item Sociocultural Internalization of Appearance Questionnaire for Adolescents, The Physical Appearance Comparison Scale, The 8-item Weight Satisfaction subscale of the Body-Esteem Scale for Adolescents and Adults, The 7-item Drive for Thinness subs |
| **Report gender differences** | Not relevant |
| **Main findings** | Elevated appearance exposure, but not overall FB usage, was significantly correlated with weight dissatisfaction, drive for thinness, thin ideal internalization, and self-objectification. Implications for eating disorder prevention programs and best practices in researching SNSs are discussed. |

# Study # 24: **«**The role of online social networking on deliberate self-harm and suicidality in adolescents: A systematized review of literature**»**

| **Title** | The role of online social networking on deliberate self-harm and suicidality in adolescents: A systematized review of literature |
| --- | --- |
| **Authors** | Memon, A. M., Sharma, S. G., Mohite, S. S., Jain, S. |
| **Year** | 2018 |
| **Journal name** | Indian Journal of Psychiatry |
| **Aims** | The specific aim is to search for the evidence of the negative influence of social media use on adolescent deliberate self-harm and suicidality. The study findings can be utilized to better create awareness in “digital native” adolescents and “digital immigrant” caregivers including, parents, schoolteachers, and physicians about negative impacts of social media and to develop protective interventions at home, in schools, and in the community. |
| **Study design** | Systematic review |
| **Study setting** | Varied |
| **Participants** | Varied |
| **Gender distribution** | Varied |
| **Type of social media use** | Studies examining the association of the use of various social networking websites including Facebook, Snapchat, Instagram, Twitter, etc., with deliberate self-harm or suicidality in adolescents. |
| **How social media was assessed** | Varied |
| **Mental Health or well-being measure** | Studies focusing on deliberate self-harm, self-injury, self-mutilation, suicidal ideation, suicide attempts, or suicide in adolescents |
| **Type of scales used** | Varied |
| **Report gender differences** | Varied |
| **Main findings** | Greater time spent on social networking websites led to higher psychological distress, an unmet need for mental health support, poor self-rated mental health, and increased suicidal ideation. |

# Study # 25: **«**Which health-related problems are associated with problematic video-gaming or social media use in adolescents? A large-scale cross-sectional study**»**

| **Title** | Which health-related problems are associated with problematic video-gaming or social media use in adolescents? A large-scale cross-sectional study |
| --- | --- |
| **Authors** | Merelle, S. Y. M., Kleiboer, A. M., Schotanus, M., Cluitmans, T. L. M., Waardenburg, C. M., Kramer, D., van de Mheen, D., van Rooij, A. J. |
| **Year** | 2017 |
| **Journal name** | Clinical Neuropsychiatry |
| **Aims** | The present study aims to identify which health-related problems are most important for adolescents that are at risk for problematic video-gaming or social media use |
| **Study design** | Cross-sectional |
| **Study setting** | School setting |
| **Participants** | 21053 |
| **Gender distribution** | 49 % males |
| **Type of social media use** | Video-gaming, social networking sites or instant messengers (e.g. Facebook, Skype, Ping, Whatsapp, Twitter) |
| **How social media was assessed** | Compulsive Internet Use Scale (CIUS) |
| **Mental Health or well-being measure** | Psychosocial problems, suicidality, bullying, major life-events, substance use |
| **Type of scales used** | Compulsive Internet Use Scale (CIUS), 25-item Strengths and Difficulties Questionnaire (SDQ), self-developed questions on general health, lifestyle and substance use. |
| **Report gender differences** | Yes |
| **Main findings** | Most mental health problems were consistently associated with both problematic video-gaming and problematic social media use, though associations were only practically relevant for conduct problems )both groups), suicidal thoughts (problematic video-gaming) and hyperactivity (problematic social media use). |

# Study # 26: **«**Social networking site use: Linked to adolescents' social self-concept, self-esteem, and depressed mood**»**

| **Title** | Social networking site use: Linked to adolescents' social self-concept, self-esteem, and depressed mood |
| --- | --- |
| **Authors** | Neira, C. J., Barber, B. L. |
| **Year** | 2014 |
| **Journal name** | Australian Journal of Psychology |
| **Aims** | The aim of this research was to investigate whether there was a relationship between adolescents’ use of social networking sites (SNSs) and their social self-concept, self-esteem, and depressed mood |
| **Study design** | Cross-sectional |
| **Study setting** | School setting |
| **Participants** | 1819 |
| **Gender distribution** | 45 % males |
| **Type of social media use** | SNS use, frequency and investment |
| **How social media was assessed** | Self-developed measures |
| **Mental Health or well-being measure** | Social self-concept, self-esteem, depressed mood |
| **Type of scales used** | Self-developed measures |
| **Report gender differences** | Yes |
| **Main findings** | The results showed that frequency of SNS use was linked to higher social self-concept while investment in SNSs was associated with lower self-esteem and higher depressed mood. Furthermore, having an SNS was linked to more negative indicators for female adolescents compared with male adolescents, although the link between frequency of use and investment in SNSs to indicators of adjustment was not moderated by gender |

# Study # 27: **«**Friends' alcohol-related social networking site activity predicts escalations in adolescent drinking: Mediation by peer norms**»**

| **Title** | Friends' alcohol-related social networking site activity predicts escalations in adolescent drinking: Mediation by peer norms |
| --- | --- |
| **Authors** | Nesi, J., Rothenberg, W. A., Hussong, A. M., Jackson, K. M. |
| **Year** | 2017 |
| **Journal name** | Journal of Adolescent Health |
| **Aims** | This study examined longitudinal associations among adolescents’ exposure to friends’ alcohol-related social networking site (SNS) postings, alcohol-favorable peer injunctive norms, and initiation of drinking behaviors. |
| **Study design** | Longitudinal |
| **Study setting** | School setting |
| **Participants** | 658 |
| **Gender distribution** | 41 % males |
| **Type of social media use** | Facebook and other social network sites |
| **How social media was assessed** | Exposure to friends’ SNS alcohol content, SNS alcohol content posted by self, time on Facebook. |
| **Mental Health or well-being measure** | Alcohol use, extreme peer orientation, peer injunctive norms |
| **Type of scales used** | Self-developed scale on social network sites and peer injunctive norms, The four-item Extreme Peer Orientation scale, The nine-item Parental Monitoring Scale |
| **Report gender differences** | No |
| **Main findings** | Exposure to peers’ alcohol-related SNS content predicted adolescents’ initiation of drinking 1 year later. Adolescents’ beliefs that peers approve of alcohol use may act as one mechanism by which exposure to friends’ alcohol-related SNS content leads to initiation of drinking behaviors. |

# Study # 28: **«**Association between insomnia and social network site use in Indonesian adolescents**»**

| **Title** | Association between insomnia and social network site use in Indonesian adolescents |
| --- | --- |
| **Authors** | Nursalam, N., Octavia, M., Tristiana, R. D., Efendi, F. |
| **Year** | 2018 |
| **Journal name** | Nursing Forum |
| **Aims** | This study aimed to explain the correlations among family support, academic stress, social network site (SNS) use, and insomnia in adolescents |
| **Study design** | Cross-sectional |
| **Study setting** | School setting |
| **Participants** | 180 |
| **Gender distribution** | 35 % males |
| **Type of social media use** | SNS type, frequency, duration, addiction |
| **How social media was assessed** | The questionnaire of social media sites addiction, adapted from Can and Kaya and from Kuss and Griffith |
| **Mental Health or well-being measure** | Insomnia, family support, school load |
| **Type of scales used** | The questionnaire of social media sites addiction, adapted from Can and Kaya and from Kuss and Griffith. The questionnaire of family support was adapted from Friedman theory. The questionnaire of insomnia was adapted from the DSM-5 |
| **Report gender differences** | No |
| **Main findings** | The reasons of SNS usage, duration of SNS usage, and academic stress are major factors associated with insomnia in adolescents. These components should be embedded in multicomponent educational intervention addressed to both adolescents and parents to reduce insomnia |

# Study # 29: **«**Negative consequences from heavy social networking in adolescents: The mediating role of fear of missing out**»**

| **Title** | Negative consequences from heavy social networking in adolescents: The mediating role of fear of missing out |
| --- | --- |
| **Authors** | Oberst, U., Wegmann, E., Stodt, B., Brand, M., Chamarro, A. |
| **Year** | 2017 |
| **Journal name** | Journal of Adolescence |
| **Aims** | We analyze the role of fear of missing out (FOMO) and intensity of SNS use for explaining the link between psychopathological symptoms and negative consequences of social networking sites (SNS) use via mobile devices |
| **Study design** | Cross-sectional |
| **Study setting** | Home setting |
| **Participants** | 1468 |
| **Gender distribution** | 377 males |
| **Type of social media use** | Social network intensity |
| **How social media was assessed** | To assess the negative consequences of mobile device use, the Spanish Cuestionario de Experiencias Relacionadas con el Movil (CERM) was used |
| **Mental Health or well-being measure** | Psychopathological symptoms, fear of missing out, negative consequences of mobile device use |
| **Type of scales used** | Hospital Anxiety and Depression Scale (HADS), the Social Networking Intensity scale (SNI), the FOMO scale |
| **Report gender differences** | Yes |
| **Main findings** | Using structural equation modeling, it was found that both FOMO and SNI mediate the link between psychopathology and CERM, but by different mechanisms. Additionally, for girls, feeling depressed seems to trigger higher SNS involvement. For boys, anxiety triggers higher SNS involvement |

# Study # 30: **«**Is social media bad for mental health and wellbeing? Exploring the perspectives of adolescents**»**

| **Title** | Is social media bad for mental health and wellbeing? Exploring the perspectives of adolescents |
| --- | --- |
| **Authors** | O'Reilly, M., Dogra, N., Whiteman, N., Hughes, J., Eruyar, S., Reilly, P. |
| **Year** | 2018 |
| **Journal name** | Clinical Child Psychology & Psychiatry |
| **Aims** | The aim of this paper is to investigate empirically how social media is viewed in terms of mental wellbeing by adolescents themselves. This aim is addressed by the question, ‘What do adolescents think of social media and its relevance to mental health and emotional wellbeing?’ |
| **Study design** | Focus group interviews |
| **Study setting** | Not mentioned |
| **Participants** | 54 |
| **Gender distribution** | Not mentioned |
| **Type of social media use** | Not specified |
| **How social media was assessed** | Not specified |
| **Mental Health or well-being measure** | Not specified |
| **Type of scales used** | None |
| **Report gender differences** | No |
| **Main findings** | It seems that adolescents need educating about the meanings of mental health and wellbeing, as well as about mental illness and the ways of preventing this or managing it if they are diagnosed with a condition. |

# Study # 31: **«**A large-scale test of the goldilocks hypothesis**»**

| **Title** | A large-scale test of the goldilocks hypothesis |
| --- | --- |
| **Authors** | Przybylski, A. K., Weinstein, N. |
| **Year** | 2017 |
| **Journal name** | Psychological Science |
| **Aims** | The goal of the present research was to evaluate different ways of understanding how screen time is linked to mental well-being, and to empirically quantify and define moderate engagement in digital activities. |
| **Study design** | Cross-sectional |
| **Study setting** | Not mentioned |
| **Participants** | 120115 |
| **Gender distribution** | Does not say |
| **Type of social media use** | Using smartphones for social networking or chatting online |
| **How social media was assessed** | Using smartphones for social networking or chatting online - not otherwise specified |
| **Mental Health or well-being measure** | Mental well-being |
| **Type of scales used** | The Warwick-edinburgh mental well-being scale |
| **Report gender differences** | Yes |
| **Main findings** | Overall, the evidence indicated that moderate use of digital technology is not intrinsically harmful and may be advantageous in a connected world. The findings inform recommendations for limiting adolescents’ technology use and provide a template for conducting rigorous investigations into the relations between digital technology and children’s and adolescents’ health. |

# Study # 32: **«**Use of social networking sites and alcohol consumption among adolescents**»**

| **Title** | Use of social networking sites and alcohol consumption among adolescents |
| --- | --- |
| **Authors** | Sampasa-Kanyinga, H., Chaput, J. P. |
| **Year** | 2016 |
| **Journal name** | Public Health |
| **Aims** | The purpose of this study was to examine the association between the use of social networking sites (SNSs) and alcohol consumption among Canadian middle and high school students, and to test whether this link varies by sex and drinking frequency or intensity. |
| **Study design** | Cross-sectional |
| **Study setting** | School setting |
| **Participants** | 10072 |
| **Gender distribution** | 4539 males |
| **Type of social media use** | Social media websites such as Facebook, Twitter, Instagram, MySpace, either posting or browsing. |
| **How social media was assessed** | Students were asked how many hours a day they usuallyspend on social media websites |
| **Mental Health or well-being measure** | Alcohol use |
| **Type of scales used** | Self-developed questionnaire |
| **Report gender differences** | Yes |
| **Main findings** | Results provide evidence that the use of SNSs is associated with alcohol consumption among adolescents. Differences between males and females in the reported associations warrant further investigations. |

# Study # 33: **«**Use of social media is associated with short sleep duration in a dose-response manner in students aged 11 to 20 years**»**

| **Title** | Use of social media is associated with short sleep duration in a dose-response manner in students aged 11 to 20 years |
| --- | --- |
| **Authors** | Sampasa-Kanyinga, H., Hamilton, H. A., Chaput, J. P. |
| **Year** | 2018 |
| **Journal name** | Acta Paediatrica |
| **Aims** | This study examined the association between social media and sleep duration among Canadian students aged 11–20. |
| **Study design** | Cross-sectional |
| **Study setting** | School setting |
| **Participants** | 5242 |
| **Gender distribution** | 51 % males |
| **Type of social media use** | Browsing or posting on social media websites such as Facebook, Twitter, MySpace and Instagram |
| **How social media was assessed** | Students were asked how many hours a day they usually spent posting or browsing on social media websites, such as Facebook, Twitter, Instagram and MySpace. |
| **Mental Health or well-being measure** | Sleep, substance use, self-reported mental health |
| **Type of scales used** | The item measuring sleep was derived from the Centers for Disease Control and Prevention’s 2013 Youth Risk Behavior Survey, other self-developed measures |
| **Report gender differences** | Yes |
| **Main findings** | Greater use of social media was associated with shorter sleep duration in a dose–response fashion among Canadian students aged 11–20. |

# Study # 34: **«**Frequent use of social networking sites is associated with poor psychological functioning among children and adolescents**»**

| **Title** | Frequent use of social networking sites is associated with poor psychological functioning among children and adolescents |
| --- | --- |
| **Authors** | Sampasa-Kanyinga, H., Lewis, R. F. |
| **Year** | 2015 |
| **Journal name** | Cyberpsychology, behavior and social networking |
| **Aims** | This study investigated the association between time spent on social networking sites (SNSs) and unmet need for mental health support, poor self-rated mental health, and reports of psychological distress and suicidal ideation in a representative sample of middle and high school children in Ottawa, Canada. |
| **Study design** | Cross-sectional |
| **Study setting** | School setting |
| **Participants** | 753 |
| **Gender distribution** | 45 % males |
| **Type of social media use** | Browsing or posting on social media websites such as Facebook, Twitter, MySpace and Instagram |
| **How social media was assessed** | Students were asked how many hours a  day they usually spend on social media Web sites such as  Facebook, Twitter, MySpace, and Instagram, either posting  or browsing |
| **Mental Health or well-being measure** | Self-reported mental health, unmet need for mental health support, psychological distress, suicidal ideation |
| **Type of scales used** | The Kessler Psychological Distress Scale (K-10), self-developed scales of SNS use, self-reported mental health, unmet need for mental health support, suicidal ideation. |
| **Report gender differences** | Yes |
| **Main findings** | Students who reported unmet need for mental health support were more likely to report using SNSs for more than 2 hours every day than those with no identified unmet need for mental health support. Daily SNS use of more than 2 hours was also independently associated with poor self-rating of mental health and experiences of high levels of psychological distress and suicidal ideation. |

# Study # 35: **«**Fear of missing out and sleep: Cognitive behavioural factors in adolescents' nighttime social media use**»**

| **Title** | Fear of missing out and sleep: Cognitive behavioural factors in adolescents' nighttime social media use |
| --- | --- |
| **Authors** | Scott, H., Woods, H. C. |
| **Year** | 2018 |
| **Journal name** | Journal of Adolescence |
| **Aims** | This study examines links between adolescents' social media habits, fear of missing out and sleep outcomes, using path analysis to evaluate a model of proposed underlying mechanisms |
| **Study design** | Cross-sectional |
| **Study setting** | School setting |
| **Participants** | 101 |
| **Gender distribution** | 34 % males |
| **Type of social media use** | Nighttime social media use |
| **How social media was assessed** | Nighttime-specific social media use was measured using a self-report questionnaire |
| **Mental Health or well-being measure** | Sleep habits, pre-sleep cognitive arousal, fear of missing out |
| **Type of scales used** | The Fear of Missing Out scale, The cognitive subscale of the Pre-Sleep Arousal Scale, self-developed questionnaires for nighttime social media use and sleep habits |
| **Report gender differences** | Yes |
| **Main findings** | Nighttime social media use was associated with later bedtimes, increased pre-sleep cognitive arousal, longer sleep onset latency and shorter sleep duration. Path analysis supported a model whereby fear of missing out predicted shorter sleep duration via two distinct mechanisms: (1) at a behavioural level, by driving late night social media use, which delays bedtimes; (2) at a cognitive level, by increasing pre-sleep cognitive arousal, thus further delaying sleep onset. |

# Study # 36: **«**The interplay between ADHD symptoms and time perspective in addictive social media use: A study on adolescent Facebook users**»**

| **Title** | The interplay between ADHD symptoms and time perspective in addictive social media use: A study on adolescent Facebook users |
| --- | --- |
| **Authors** | Settanni, M., Marengo, D., Fabris, M. A., Longobardi, C. |
| **Year** | 2018 |
| **Journal name** | Children and Youth Services Review |
| **Aims** | The present study investigates the interplay between ADHD symptoms and time perspective in promoting addictive Facebook use in a sample of adolescent Facebook users |
| **Study design** | Cross-sectional |
| **Study setting** | School setting |
| **Participants** | 283 |
| **Gender distribution** | 50 % males |
| **Type of social media use** | Addictive Facebook use |
| **How social media was assessed** | Bergen Facebook Addiction Scale |
| **Mental Health or well-being measure** | ADHD symptoms, addictive facebook use, time perspective (how one relates to past experiences and future consequences) |
| **Type of scales used** | Bergen Facebook Addiction Scale, Zimbardo Time Perspective Inventory (ZTPI) for adolescents, ADHD subscaleof the Italian self-report version of the Strength and Difficulties Questionnaires |
| **Report gender differences** | Yes |
| **Main findings** | Results of regression analyses showed that ADHD symptoms positively predicted addictive Facebook use, past negative and present fatalistic orientation, and negatively predicted future orientation (ZTPI). Further, past negative and present fatalistic TP orientations acted as mediators of the relationship between ADHD symptoms and addictive Facebook use: ADHD symptoms favor an increase in problematic use of social media, and this effect appears to be linked to a worsening in the perception of past and present experiences (ZTPI) |

# Study # 37: **«**Media use is linked to lower psychological well-being: Evidence from three datasets**»**

| **Title** | Media use is linked to lower psychological well-being: Evidence from three datasets |
| --- | --- |
| **Authors** | Twenge, J. M., Campbell, W. K. |
| **Year** | 2019 |
| **Journal name** | Psychiatric Quarterly |
| **Aims** | By analyzing all three datasets using the same analytic techniques, we aim to provide a comprehensive view of the association between digital media use and psychological well-being and add insights to the debate surrounding the shape and size of the effects in this area. We also aim to address whether effect sizes comparing well-being between light and moderate digital media use differ from those comparing moderate use with heavy use, a question not addressed in previous analyses. |
| **Study design** | Cross-sectional |
| **Study setting** | Not mentioned |
| **Participants** | 221096 |
| **Gender distribution** | Not mentioned |
| **Type of social media use** | Digital media use (playing games, social networking, chatting) |
| **How social media was assessed** | Frequency and amount of time used on social media |
| **Mental Health or well-being measure** | Well-being |
| **Type of scales used** | The 14-item Warwick-Edinburgh Mental Well-Being Scale |
| **Report gender differences** | No (?) |
| **Main findings** | Heavy users (vs. light) of digital media were 48% to 171% more likely to be unhappy, to be in low in well-being, or to have suicide risk factors such as depression, suicidal ideation, or past suicide attempts. Heavy users (vs. light) were twice as likely to report having attempted suicide. Light users (rather than non- or moderate users) were highest in well-being, and for most digital media use the largest drop in well-being occurred between moderate use and heavy use |

# Study # 38: **«**The impact of heavy and disordered use of games and social media on adolescents' psychological, social, and school functioning**»**

| **Title** | The impact of heavy and disordered use of games and social media on adolescents' psychological, social, and school functioning |
| --- | --- |
| **Authors** | van den Eijnden, R., Koning, I., Doornwaard, S., van Gurp, F., Ter Bogt, T. |
| **Year** | 2018 |
| **Journal name** | Journal of Behavioral Addictions |
| **Aims** | To extend the scholarly debate on (a) whether or not the compulsive use of games and social media should be regarded as behavioral addictions and (b) whether the nine DSM-5 criteria for Internet gaming disorder are appropriate to distinguish highly engaged, non-disordered users of games and social media from disordered user. This study investigated the impact of engaged and disordered use of games and social media on the psychosocial well-being and school performances of adolescents. |
| **Study design** | Longitudinal |
| **Study setting** | School setting |
| **Participants** | 538 |
| **Gender distribution** | 49 % males |
| **Type of social media use** | Gaming, social media, social media disorder |
| **How social media was assessed** | Frequency of use, type of use, social media disorder |
| **Mental Health or well-being measure** | Social media disorder, internet gaming disorder, life satisfaction |
| **Type of scales used** | Internet Gaming Disorder scale, 5-item Satisfaction with Life Scale, Harters’ Self Perception Profile of Adolescents, self-developed measures |
| **Report gender differences** | Yes |
| **Main findings** | The findings propose that symptoms of disordered use of games and social media predict a decrease in the psychosocial well-being and school performances of adolescents, thereby meeting one of the core criteria of behavioral addictions. |

# Study # 39: **«**Social networking sites addiction and adolescent depression: A moderated mediation model of rumination and self-esteem**»**

| **Title** | Social networking sites addiction and adolescent depression: A moderated mediation model of rumination and self-esteem |
| --- | --- |
| **Authors** | Wang, P., Wang, X., Yingqiu, W., Xie, X., Wang, X., Zhao, F., Ouyang, M. K., Lei, L. |
| **Year** | 2018 |
| **Journal name** | Personality and Individual Differences |
| **Aims** | The present study examined whether rumination mediated therelation between social networking site (SNS) addiction and depression, and whether the mediating effect was moderated by self-esteem. |
| **Study design** | Cross-sectional |
| **Study setting** | School setting |
| **Participants** | 365 |
| **Gender distribution** | 48 % males |
| **Type of social media use** | Social networking sites addicton |
| **How social media was assessed** | The Facebook Intrusion Questionnaire |
| **Mental Health or well-being measure** | Depression, rumination, self-esteem |
| **Type of scales used** | The Facebook Intrusion Questionnaire, Ruminative Response Scale, Center for Epidemiological Studies Depression Scale, Rosenberg Self-Esteem Scale |
| **Report gender differences** | No |
| **Main findings** | The results indicated that SNS addiction was positively associated with depression. Mediation analysis indicated that rumination mediated the relation between SNS addiction and depression. Moderated mediated analysis further revealed that the path between rumination and depression was stronger for individuals with lower self-esteem than individuals with higher self-esteem |

# Study # 40: **«**#Sleepyteens: Social media use in adolescence is associated with poor sleep quality, anxiety, depression and low self-esteem**»**

| **Title** | #Sleepyteens: Social media use in adolescence is associated with poor sleep quality, anxiety, depression and low self-esteem |
| --- | --- |
| **Authors** | Woods, H. C., Scott, H. |
| **Year** | 2016 |
| **Journal name** | Journal of Adolescence |
| **Aims** | This study examined how social media use related to sleep quality, self-esteem, anxiety and depression in 467 Scottish adolescents. We measured overall social media use, nighttime-specific social media use, emotional investment in social media, sleep quality, self-esteem and levels of anxiety and depression. |
| **Study design** | Cross-sectional |
| **Study setting** | School setting |
| **Participants** | 467 |
| **Gender distribution** | Not mentioned |
| **Type of social media use** | Emotional investment in social media, overall and nightime-specific social media use |
| **How social media was assessed** | Frequency, duration, number of sites, spread throughout the day, |
| **Mental Health or well-being measure** | Sleep, anxiety, depression, self-esteem |
| **Type of scales used** | Pittsburgh Sleep Quality Index, The Hospital Anxiety and Depression Scale, The Rosenberg Self-Esteem Scale, The Social Integration and EmotionalConnection subscale of the Social Media Use Integration Scale, self-developed measure of overall and nighttime-specific social media use |
| **Report gender differences** | Not relevant |
| **Main findings** | Adolescents who used social media more both overall and at night and those who were more emotionally invested in social media experienced poorer sleep quality, lower self-esteem and higher levels of anxiety and depression. Nighttime-specific social media use predicted poorer sleep quality after controlling for anxiety, depression and self-esteem. |

# Study # 41: **«**Online communication, social media and adolescent wellbeing: A systematic narrative review**»**

| **Title** | Online communication, social media and adolescent wellbeing: A systematic narrative review |
| --- | --- |
| **Authors** | Best, P., Manktelow, R., Taylor, B. |
| **Year** | 2014 |
| **Journal name** | Children and Youth Services Review |
| **Aims** | To systematically review and synthesise current empirical research on this topic, identifying both the beneficial and harmful effects of online communication and social media technology amongst young people. |
| **Study design** | A systematic narrative review |
| **Study setting** | Varied |
| **Participants** | Varied |
| **Gender distribution** | Varied |
| **Type of social media use** | Varied |
| **How social media was assessed** | Varied |
| **Mental Health or well-being measure** | Well-being issues or related concepts |
| **Type of scales used** | Varied |
| **Report gender differences** | Yes |
| **Main findings** | This systematic narrative review has revealed contradictory evidence while revealing an absence of robust causal research regarding the impact of social media on mental wellbeing of young people. Online technologies are increasingly being used for health and social care purposes, but further research is required to give confidence that these are appropriately designed to promote the mental health care and support of young people |

# Study # 42: **«**Gender differences in the associations between age trends of social media interaction and well-being among 10-15 year olds in the UK**»**

| **Title** | Gender differences in the associations between age trends of social media interaction and well-being among 10-15 year olds in the UK |
| --- | --- |
| **Authors** | Booker, C. L., Kelly, Y. J., Sacker, A. |
| **Year** | 2018 |
| **Journal name** | BMC Public Health |
| **Aims** | The aim of this study was to examine whether the changes in social media interaction and two well-being measures are related across ages using parallel growth models. |
| **Study design** | Longitudinal |
| **Study setting** | Home setting |
| **Participants** | 9859 |
| **Gender distribution** | 51 % males |
| **Type of social media use** | Bebo, Facebook, Myspace |
| **How social media was assessed** | Participation and amount of use |
| **Mental Health or well-being measure** | Well-being |
| **Type of scales used** | Strengths and Difficulties Questionnaire (SDQ), self-developed questionnaires |
| **Report gender differences** | Yes |
| **Main findings** | High levels of social media interaction in early adolescence have implications for well-being in later adolescence, particularly for females. The lack of an association among males suggests other factors might be associated with their reduction in well-being with age. These findings contribute to the debate on causality and may inform future policy and interventions. |

# Study # 43: **«**Adolescents' social network site use, peer appearance-related feedback, and body dissatisfaction: Testing a mediation model**»**

| **Title** | Adolescents' social network site use, peer appearance-related feedback, and body dissatisfaction: Testing a mediation model |
| --- | --- |
| **Authors** | de Vries, D. A., Peter, J., de Graaf, H., Nikken, P. |
| **Year** | 2016 |
| **Journal name** | Journal of Youth & Adolescence |
| **Aims** | Previous correlational research indicates that adolescent girls who use social network sites more frequently are more dissatisfied with their bodies. However, we know little about the causal direction of this relationship, the mechanisms underlying this relationship, and whether this relationship also occurs among boys to the same extent. The present two-wave panel study (18 month time lag) among 604 Dutch adolescents (aged 11–18; 50.7 % female; 97.7 % native Dutch) aimed to fill these gaps in knowledge |
| **Study design** | Longitudinal |
| **Study setting** | Home setting |
| **Participants** | 604 |
| **Gender distribution** | 49 % males |
| **Type of social media use** | Hyves.nl, Myspace, Facebook etc. |
| **How social media was assessed** | Frequency of use last 6 months |
| **Mental Health or well-being measure** | Body dissatisfaction, BMI |
| **Type of scales used** | The Body Areas Satisfaction Scale, self-developed scales |
| **Report gender differences** | Yes |
| **Main findings** | Structural equation modeling showed that social network site use predicted increased body dissatisfaction and increased peer influence on body image in the form of receiving peer appearance-related feedback. Peer appearance-related feedback did not predict body dissatisfaction and thus did not mediate the effect of social network site use on body dissatisfaction. Gender did not moderate the findings. Hence, social network sites can play an adverse role in the body image of both adolescent boys and girls |

# Study # 44: **«**Concurrent and prospective analyses of peer, television and social media influences on body dissatisfaction, eating disorder symptoms and life satisfaction in adolescent girls**»**

| **Title** | Concurrent and prospective analyses of peer, television and social media influences on body dissatisfaction, eating disorder symptoms and life satisfaction in adolescent girls |
| --- | --- |
| **Authors** | Ferguson, C. J., Muñoz, M. E., Garza, A., Galindo, M. |
| **Year** | 2014 |
| **Journal name** | Journal of Youth & Adolescence |
| **Aims** | The current study examines television, social media and peer competition influences on body dissatisfaction, eating disorder symptoms and life satisfaction in a sample of 237 mostly Hispanic girls. |
| **Study design** | Cross-sectional |
| **Study setting** | Not mentioned |
| **Participants** | 237 |
| **Gender distribution** | 0 males |
| **Type of social media use** | Most common social media sites, social gaming, MMOs |
| **How social media was assessed** | Frequency of most common social media |
| **Mental Health or well-being measure** | Body Image Dissatisfaction, Eating disorder symptoms, life satisfaction, depressive symptoms, peer competition |
| **Type of scales used** | Female Competition Stress Test, The Body Esteem Scale for Adolescents and Adults, Eating Attitudes Test, Life satisfaction, Beck Anxiety Inventory, Zung Depression Inventory, Child Behavior Checklist, Parenting Styles Questionnaire, Family Conflict Scale, |
| **Report gender differences** | Not relevant |
| **Main findings** | It is concluded that the negative influences of social comparison are focused on peers rather than television or social media exposure |

# Study # 45: **«**Bullying and cyberbullying studies in the school-aged population on the island of Ireland: A meta-analysis**»**

| **Title** | Bullying and cyberbullying studies in the school-aged population on the island of Ireland: A meta-analysis |
| --- | --- |
| **Authors** | Foody, M., Samara, M., O'Higgins, N. J. |
| **Year** | 2017 |
| **Journal name** | British Journal of Educational Psychology |
| **Aims** | We conducted a meta-analysis of traditional and cyberbullying studies in the Republic and North of Ireland to gain an understanding of prevalence rates and associated issues (particularly psychological correlates and intervention strategies) among young people (primary and secondary school students). |
| **Study design** | Meta-analysis |
| **Study setting** | Varied |
| **Participants** | Varied |
| **Gender distribution** | Varied |
| **Type of social media use** | Varied |
| **How social media was assessed** | Varied |
| **Mental Health or well-being measure** | Varied |
| **Type of scales used** | Varied |
| **Report gender differences** | Varied |
| **Main findings** | The results demonstrate the influence moderating factors (e.g., assessment tools, answer scale, time frame) have on reported prevalence rates. These results are discussed in light of current studies, and points for future research are considered. |

# Study # 46: **«**The reciprocal associations between sharing alcohol references on social networking sites and binge drinking: A longitudinal study among late adolescents**»**

| **Title** | The reciprocal associations between sharing alcohol references on social networking sites and binge drinking: A longitudinal study among late adolescents |
| --- | --- |
| **Authors** | Geusens, F., Beullens, K. |
| **Year** | 2017 |
| **Journal name** | Computers in Human Behavior |
| **Aims** | The present longitudinal study (N 1006) adds to this line of research by examining the reciprocity of sharing alcohol references on social media and binge drinking among late adolescents (age 16-20 at baseline). |
| **Study design** | Longitudinal |
| **Study setting** | School setting |
| **Participants** | 1006 |
| **Gender distribution** | 51 % males at baseline and 40 % at follow-up |
| **Type of social media use** | Sharing alcohol references on social network sites |
| **How social media was assessed** | Photos or video clips, textual updates concerning alcohol, both open sharing and private sharing |
| **Mental Health or well-being measure** | Binge drinking, perceived feedback from peers |
| **Type of scales used** | Self-developed questionnaires |
| **Report gender differences** | Yes |
| **Main findings** | Overall, the results suggest that binge drinking and alcohol-related self-presentation on social media should be studied as tied behaviors, affecting each other simultaneously over time. |

# Study # 47: **«**Comparing personality traits, mental health and self-esteem in users and non-users of social networks**»**

| **Title** | Comparing personality traits, mental health and self-esteem in users and non-users of social networks |
| --- | --- |
| **Authors** | Jafarpour, J., Jadidi, H., Almadani, S. A. H. |
| **Year** | 2017 |
| **Journal name** | Razavi International Journal of Medicine |
| **Aims** | This study aimed to examine the personality traits, mental health and self-esteem in users and non-users of social networks. |
| **Study design** | Casual-comparative |
| **Study setting** | Not mentioned |
| **Participants** | 372 |
| **Gender distribution** | 0 % males |
| **Type of social media use** | General social networks |
| **How social media was assessed** | Duration of the use of social networks |
| **Mental Health or well-being measure** | Mental health, self-esteem |
| **Type of scales used** | Rosenberg self-esteem scale, Goldberg Mental Health Questionnaire |
| **Report gender differences** | Not relevant |
| **Main findings** | The findings of this study emphasize the attention to psychological factors in planning of preventive interventions. |

# Study # 48: **«**The double meaning of online social space: Three-way interactions among social anxiety, online social behavior, and offline social behavior**»**

| **Title** | The double meaning of online social space: Three-way interactions among social anxiety, online social behavior, and offline social behavior |
| --- | --- |
| **Authors** | Koo, H. J., Woo, S., Yang, E., Kwon, J. H. |
| **Year** | 2015 |
| **Journal name** | Cyberpsychology, Behavior, and Social Networking |
| **Aims** | The present study aimed to investigate how online and offline social behavior interact with each other ultimately to affect the well-being of socially anxious adolescents |
| **Study design** | Cross-sectional |
| **Study setting** | Home setting |
| **Participants** | 656 |
| **Gender distribution** | 48 % males |
| **Type of social media use** | General social networks |
| **How social media was assessed** | Online relational maintenance behaviors |
| **Mental Health or well-being measure** | Social anxiety, well-being |
| **Type of scales used** | Korean-Social Avoidance and Distress Scale, Korean version of the Relational Maintenance Behavior Questionnaire, the Korean  validated Mental Health Continuum Short Form |
| **Report gender differences** | Yes |
| **Main findings** | First, online social behavior was associated with lower well-being of adolescents with higher social anxiety under conditions of low engagement in offline social behavior. In contrast, a higher level of online social behavior predicted greater well-being for individuals with high social anxiety under conditions of more engagement in offline social behavior. Second, online social behavior was not significantly related to well-being in youths with low social anxiety under conditions of both high and low engagement in offline social behavior. |

# Study # 49: **«**The role of online social network chatting for alcohol use in adolescence: Testing three peer-related pathways in a Swedish population-based sample**»**

| **Title** | The role of online social network chatting for alcohol use in adolescence: Testing three peer-related pathways in a Swedish population-based sample |
| --- | --- |
| **Authors** | Larm, P. A., Åslund, C., Nilsson, K. W. |
| **Year** | 2017 |
| **Journal name** | Computers in Human Behavior |
| **Aims** | The aim of the study was to examine whether online social network chatting (OSNC) is related to any of three peer-related pathways to alcohol use among adolescents including a stress-exposure pathway, a peer status pathway and a social context pathway. |
| **Study design** | Cross-sectional |
| **Study setting** | School setting |
| **Participants** | 2439 |
| **Gender distribution** | 48 % males |
| **Type of social media use** | Myspace, Facebook and others |
| **How social media was assessed** | Frequency of use, amount of time per use |
| **Mental Health or well-being measure** | Stress-exposure, peer status, alcohol use, depressive symptoms, bullying |
| **Type of scales used** | Two questions about frequency and amount of time of engaging in OSNC, with a summarized score varying from 0 to 10 |
| **Report gender differences** | Yes |
| **Main findings** | First, OSNC had only a marginal role for two of the three stipulated peer-related pathways to alcohol use among adolescents. Second, adolescents exposed to stressors with elevated OSNC had lower alcohol use, which may indicate that OSNC is a promising tool in the prevention work for this group. Third, the social context pathway was most important for adolescents' alcohol use and wasespecially important for those with high scores on OSNC and for girls. Fourth, the association between OSNC and alcohol use in adolescence is due mostly to factors other than those that are peer related, which calls for research on underlying biological and psychological factors |

# Study # 50: **«**Qzone use and depression among Chinese adolescents: A moderated mediation model**»**

| **Title** | Qzone use and depression among Chinese adolescents: A moderated mediation model |
| --- | --- |
| **Authors** | Niu, G. F., Luo, Y. J., Sun, X. J., Zhou, Z. K., Yu, F., Yang, S. L., Zhao, L. |
| **Year** | 2018 |
| **Journal name** | Journal of Affective Disorders |
| **Aims** | Based on social comparison theory, the current study aimed to investigate the association between Chinese adolescents’ social networking site (SNS; Qzone) use and depression, as well as the mediating role of negative social comparison and the moderating role of self-esteem. |
| **Study design** | Cross-sectional |
| **Study setting** | School setting |
| **Participants** | 764 |
| **Gender distribution** | 406 males |
| **Type of social media use** | Qzone |
| **How social media was assessed** | Amount of time on use and attitudinal questions |
| **Mental Health or well-being measure** | Negative social comparison, self-esteem, depression |
| **Type of scales used** | Eight items adapted from the Facebook intensity scale, Rosenbergs Self-esteem scale, The Chinese version of the Center for Epidemiologic Studies Depression Scale (CES-D) |
| **Report gender differences** | No |
| **Main findings** | Negative social comparison may be a key factor and mechanism accounting for the positive association between SNS use and depression, while self-esteem could protect adolescents from the adverse outcome of SNS use |

# Study # 51: **«**Impact of social media on the health of children and young people**»**

| **Title** | Impact of social media on the health of children and young people |
| --- | --- |
| **Authors** | Richards, D., Caldwell, P., Go, H. |
| **Year** | 2015 |
| **Journal name** | Journal of Paediatrics & Child Health |
| **Aims** | This paper reviews the literature on the impact of social media on the health of children and young people |
| **Study design** | Review article |
| **Study setting** | Varied |
| **Participants** | Varied |
| **Gender distribution** | Varied |
| **Type of social media use** | keywords ‘social media’ or ‘facebook’ or  ‘twitter’ or ‘youtube’ or ‘myspace’ |
| **How social media was assessed** | Varied |
| **Mental Health or well-being measure** | Self-esteem, well-being, depression, online risk-taking behaviour |
| **Type of scales used** | Varied |
| **Report gender differences** | Uncertain |
| **Main findings** | Social media is here to stay and increasingly being used by children and young people. Although social media can have a positive impact on the health and well-being of children and young people, it can also pose risks for these vulnerable populations. These risks can be minimised by appropriate website design, identification of those most at risk and development of strategies to help them manage these risks. Some of the social media risks for children can be minimised by adding in safety features; however, that is unlikely to happen in social media sites that are also used by adults because of the restrictive nature of these controls |

# Study # 52: **«**The reciprocal and indirect relationships between passive Facebook use, comparison on Facebook, and adolescents' body dissatisfaction**»**

| **Title** | The reciprocal and indirect relationships between passive Facebook use, comparison on Facebook, and adolescents' body dissatisfaction |
| --- | --- |
| **Authors** | Rousseau, A., Eggermont, S., Frison, E. |
| **Year** | 2017 |
| **Journal name** | Computers in Human Behavior |
| **Aims** | Given that no study thus far investigated whether the reciprocal and indirect relationships between passive Facebook use, comparison on Facebook, and adolescents' body dissatisfaction differ between boys and girls, the present study will explore the moderating role of gender in the hypothesized associations. |
| **Study design** | Longitudinal |
| **Study setting** | School setting |
| **Participants** | 1840 |
| **Gender distribution** | 52 % males |
| **Type of social media use** | Facebook |
| **How social media was assessed** | Passive Facebook use, comparison on Facebook, amount of time on Facebook, regularity, |
| **Mental Health or well-being measure** | Body dissatisfaction, |
| **Type of scales used** | Passive Facebook Use Subscale of the Multidimensional Scale of Facebook Use, The Body Dissatisfaction Subscale of the Body Attitude Test, self-developed questionnaires |
| **Report gender differences** | Yes |
| **Main findings** | Despite these limitations, the current findings provide valuable insights into how a specific type of Facebook use (i.e., passive Facebook use) can contribute to adolescents' body dissatisfaction. We believe that our findings extend prior research in at least two important ways. First, the present two-wave panel study demonstrated a reciprocal dynamic between passive Facebook use, comparison on Facebook, and adolescents' body dissatisfaction. Although social comparison is almost inevitable when using Facebook, the observed reciprocal pathway leads to the tentative conclusion that body dissatisfied adolescents also deliberately seek out comparison on Facebook, possibly with the aim of self-improvement. Second, we demonstrated the important role of adolescents' gender in the relationship between passive Facebook use and comparison on Facebook, as passive Facebook was positively related to comparison on Facebook over time among boys, but not among girls. |

# Study # 53: **«**The dark side of internet use: Two longitudinal studies of excessive internet use, depressive symptoms, school burnout and engagement among Finnish early and late adolescents**»**

| **Title** | The dark side of internet use: Two longitudinal studies of excessive internet use, depressive symptoms, school burnout and engagement among Finnish early and late adolescents |
| --- | --- |
| **Authors** | Salmela-Aro, K., Upadyaya, K., Hakkarainen, K., Lonka, K., Alho, K. |
| **Year** | 2017 |
| **Journal name** | Journal of Youth & Adolescence |
| **Aims** | In this study, we examined the longitudinal paths between excessive internet use, depressive symptoms, school burnout and engagement. |
| **Study design** | Longitudinal |
| **Study setting** | School setting |
| **Participants** | 1702 |
| **Gender distribution** | 47 % males |
| **Type of social media use** | The use of information and communication technologies |
| **How social media was assessed** | The urge to use social media (SOME) and if SOME causes neglect of schoolwork |
| **Mental Health or well-being measure** | School burnout, depressive symptoms, excessive internet use, |
| **Type of scales used** | School Burnout Inventory, DEPS Depression Scale, self-developed questionnaire |
| **Report gender differences** | Yes |
| **Main findings** | Emotional engagement, school burnout and depressive symptoms were found to function as distinct psychological processes each of which makes a unique contribution to adolescent excessive internet use. Furthermore, the quality of students’ emotional involvement in school may influence excessive internet use through multiple pathways |

# Study # 54: **«**Facebook and body image concern in adolescent girls: A prospective study**»**

| **Title** | Facebook and body image concern in adolescent girls: A prospective study |
| --- | --- |
| **Authors** | Tiggemann, M., Slater, A. |
| **Year** | 2017 |
| **Journal name** | International Journal of Eating Disorders |
| **Aims** | The primary aim of the study was to examine the relationship across time between Facebook use and body image concern in adolescent girls. |
| **Study design** | Cross-sectional |
| **Study setting** | School setting |
| **Participants** | 438 |
| **Gender distribution** | 0 % males |
| **Type of social media use** | Facebook |
| **How social media was assessed** | Amount of facebook-use and number of friends |
| **Mental Health or well-being measure** | Body image concerns |
| **Type of scales used** | Sociocultural Attitudes Toward Appearance Questionnaire, Body Surveillance was assessed by the Objectified Body Consciousness Scale–Youth, Drive for Thinness Scale of the Eating Disorder Inventory |
| **Report gender differences** | Not relevant |
| **Main findings** | It was concluded that Facebook “friendships” represent a potent sociocultural force in the body image of adolescent girls |

# Study # 55: **«**Internet use and web communication networks, sources of social support, and forms of suicidal and nonsuicidal self-injury among adolescents: Different patterns between genders**»**

| **Title** | Internet use and web communication networks, sources of social support, and forms of suicidal and nonsuicidal self-injury among adolescents: Different patterns between genders |
| --- | --- |
| **Authors** | Tseng, F. Y., Yang, H. J. |
| **Year** | 2015 |
| **Journal name** | Suicide and Life-Threatening Behavior |
| **Aims** | The relationships of Internet use, web communication, and sources of social support with adolescent self-injurious thoughts and behaviors (SITBs) in Taiwan were investigated. |
| **Study design** | Cross-sectional |
| **Study setting** | Not mentioned |
| **Participants** | 391 |
| **Gender distribution** | 45 % males |
| **Type of social media use** | Internet use and web communication network |
| **How social media was assessed** | Problematic Internet Use, amount, frequency, number of friends, and 16 questions on web communication networks |
| **Mental Health or well-being measure** | Self-injury, depressive symptoms, social support |
| **Type of scales used** | Self-injurious Thoughts and Behaviors Interview, the Problematic  Internet Use and Physical and Mental Health Questionnaire, The Multidimensional Scale of Perceived Social Support, the Center for Epidemiological Study-Depression Scale |
| **Report gender differences** | Yes |
| **Main findings** | Findings show that girls are more likely to have SITBs, except for suicide gestures. Web communication is a risk factor for SITBs in boys but not in girls. Family support is protective in both genders. Support from friends is protective and support from significant others was a risk factor for suicide plans in girls. Support from virtual social communities can have both positive and negative effects on adolescent SITBs, with different effects by gender. |

# Study # 56: **«**Increases in depressive symptoms, suicide-related outcomes, and suicide rates among U.S. adolescents after 2010 and links to increased new media screen time**»**

| **Title** | Increases in depressive symptoms, suicide-related outcomes, and suicide rates among U.S. adolescents after 2010 and links to increased new media screen time |
| --- | --- |
| **Authors** | Twenge, J. M., Joiner, T. E., Rogers, M. L., Martin, G. N. |
| **Year** | 2018 |
| **Journal name** | Clinical Psychological Science |
| **Aims** | The current research has two goals. First, we seek to determine if the prevalence of depressive symptoms, suicide-related outcomes (i.e., suicidal ideation, plans, and attempts), and suicide deaths has increased in nationally representative samples of U.S. adolescents in recent years and whether these birth cohort trends2 differ by gender, race, age, region, and socioeconomic status (SES). Second, we examine possible causes behind trends in adolescents’ mental health, primarily focusing on shifts in adolescents’ use of leisure time in these same nationally representative samples |
| **Study design** | Time-lag design |
| **Study setting** | Not mentioned |
| **Participants** | 506820 |
| **Gender distribution** | Not mentioned |
| **Type of social media use** | Social network sites (like Facebook) |
| **How social media was assessed** | Frequency of use |
| **Mental Health or well-being measure** | Depressive symptoms, suicide-related outcomes |
| **Type of scales used** | Bentler Medical and Psychological Functioning Inventory  depression scale, self-developed questionnaires |
| **Report gender differences** | Yes |
| **Main findings** | In conclusion, adolescent mental health issues rose sharply since 2010, especially among females. New media screen time is both associated with mental health issues and increased over this time period. Thus, it seems likely that the concomitant rise of screen time and adolescent depression and suicide is not coincidental. |

# Study # 57: **«**Associations among screen time and unhealthy behaviors, academic performance, and well-being in Chinese adolescents**»**

| **Title** | Associations among screen time and unhealthy behaviors, academic performance, and well-being in Chinese adolescents |
| --- | --- |
| **Authors** | Yan, H., Zhang, R., Oniffrey, T. M., Chen, G., Wang, Y., Wu, Y., Zhang, X., Wang, Q., Ma, L., Li, R., Moore, J. B. |
| **Year** | 2017 |
| **Journal name** | International Journal of Environmental Research & Public Health [Electronic Resource] |
| **Aims** | The aim of this study was to determine the amount of time adolescents in Wuhan, China, spent on screen-based behaviors, and the associations of this  with adiposity, unhealthy eating behaviors, sleep, physical activity, academic performance, anxiety, self-esteem, and life satisfaction |
| **Study design** | Cross-sectional |
| **Study setting** | Home setting |
| **Participants** | 2625 |
| **Gender distribution** | 53 % males |
| **Type of social media use** | Social media sites/apps, playing e-games |
| **How social media was assessed** | Amount of time on use |
| **Mental Health or well-being measure** | Sleep, anxiety, self-esteem, life-satisfaction |
| **Type of scales used** | The Middle School Student Mental Health Scale, The Satisfaction With Life Scale, The Rosenberg Self-Esteem Scale, self-developed questionnaires |
| **Report gender differences** | Yes |
| **Main findings** | We observed differential associations between time spent on screen-based behaviors and unhealthy eating behaviors, BMI z-score, academic performance, and mental health in this sample of Chinese adolescents. If these results are indeed confirmed, Chinese school officials and policymakers should establish strategies to minimize these negative effects |

# Study # 58: **«**Cyberbullying: Review of an old problem gone viral**»**

| **Title** | Cyberbullying: Review of an old problem gone viral |
| --- | --- |
| **Authors** | Aboujaoude, E., Savage, M. W., Starcevic, V. & Salame, W. O. |
| **Year** | 2015 |
| **Journal name** | Journal of Adolescent Health |
| **Aims** | The aim was to provide a comprehensive, integrative, and none theory-driven review of cyberbullying, including its history, phenomenology, epidemiological aspects, associated psychopathology, and prevention and control. |
| **Study design** | Systematic review |
| **Study setting** | Varied |
| **Participants** | Varied |
| **Gender distribution** | Varied |
| **Type of social media use** | Cyberbullying |
| **How social media was assessed** | Varied |
| **Mental Health or well-being measure** | Various psychopathology |
| **Type of scales used** | Various |
| **Report gender differences** | Yes |
| **Main findings** | More research is needed into cyberbullying, but available data suggest a serious problem whose consequences are real and should not be dismissed as a “virtual” by-product of an increasingly digitalized childhood and adolescence |

# Study # 59: **«**A longitudinal study of the social and emotional predictors and consequences of cyber and traditional bullying victimisation**»**

| **Title** | A longitudinal study of the social and emotional predictors and consequences of cyber and traditional bullying victimisation |
| --- | --- |
| **Authors** | Cross, D., Lester, L., Barnes, A. |
| **Year** | 2015 |
| **Journal name** | International Journal of Public Health |
| **Aims** | This study aimed to identify the associations between the relative contribution of cyberbullying victimisation and traditional bullying victimisation on social and emotional antecedents and outcomes among adolescents. |
| **Study design** | Longitudinal |
| **Study setting** | Home setting |
| **Participants** | 1504 |
| **Gender distribution** | 47 % males |
| **Type of social media use** | Facebook, MSN, e-mails etc |
| **How social media was assessed** | Frequency of occurence |
| **Mental Health or well-being measure** | Well-being |
| **Type of scales used** | Self-developed questionnaire |
| **Report gender differences** | Yes |
| **Main findings** | These findings suggest a high coexistence of cyber and traditional bullying behaviours and their antecedents, and higher levels of harm from a combination of these behaviours for adolescents over time |

# Study # 60: **«**Longitudinal associations between cyberbullying involvement and adolescent mental health**»**

| **Title** | Longitudinal Associations Between Cyberbullying Involvement and Adolescent Mental Health |
| --- | --- |
| **Authors** | Fahy, A. E., Stansfeld, S. A., Smuk, M., Smith, N. R., Cummins, S., Clark, C. |
| **Year** | 2016 |
| **Journal name** | Journal of Adolescent Health |
| **Aims** | Cyberbullying differs from face-to-face bullying and may negatively influence adolescentmental health, but there is a lack of definitive research on this topic. This study examines longi-tudinal associations between cyberbullying involvement and adolescent mental health. |
| **Study design** | Longitudinal |
| **Study setting** | Not mentioned |
| **Participants** | 2480 |
| **Gender distribution** | 55 % males |
| **Type of social media use** | Internet & social media |
| **How social media was assessed** | Comments or threats on internet/social media |
| **Mental Health or well-being measure** | Depression, social anxiety, well-being |
| **Type of scales used** | Cyberbullying involvement by Ybarra, Short Mood and Feelins Questionnaire, Mini Social Phobia Inventory, Warwick-Edinburgh Mental Well-Being Scale |
| **Report gender differences** | Yes |
| **Main findings** | This study emphasizes the high prevalence of cyberbullying and the potential ofcybervictimization as a risk factor for future depressive symptoms, social anxiety symptoms, andbelow average well-being among adolescents. |

# Study # 61: **«**Peer cybervictimization among adolescents and the associated internalizing and externalizing problems: A meta-analysis**»**

| **Title** | Peer cybervictimization among adolescents and the associated internalizing andeExternalizing problems: A meta-analysis |
| --- | --- |
| **Authors** | Fisher, B. W., Gardella, J. H., Teurbe-Tolon, A. R. |
| **Year** | 2016 |
| **Journal name** | Journal of Youth & Adolescence |
| **Aims** | Specifically, this study is guided by the following research questions: (a) What is the association between experiencing peer cybervictimization and internalizing problems among United States adolescents? (b) What is the association between experiencing peer cybervictimization and externalizing problems among United States adolescents? (c) Do these relationships depend on study- and sample-level characteristics? |
| **Study design** | Systematic review |
| **Study setting** | Varied |
| **Participants** | 257678 |
| **Gender distribution** | Not mentioned |
| **Type of social media use** | Varied |
| **How social media was assessed** | Varied |
| **Mental Health or well-being measure** | Internalizing problems, externalizing problems |
| **Type of scales used** | Varied |
| **Report gender differences** | Yes |
| **Main findings** | The results of a series of random effects meta-analyses using robust variance estimation indicated positive and significant relationships between peer cybervictimization and a series of internalizing and externalizing problems, with point estimates of this relationship ranging from Pearson’s r = .14 to .34. |

# Study # 62: **«**Peer victimization and suicidal ideation: The role of gender and depression in a school-based sample**»**

| **Title** | Peer victimization and suicidal ideation: The role of gender and depression in a school-based sample |
| --- | --- |
| **Authors** | Fredrick, S. S., Demaray, M. K. |
| **Year** | 2018 |
| **Journal name** | Journal of School Psychology |
| **Aims** | The current study investigated the relations among traditional and cyber victimization, depressive symptoms, suicidal ideation, and gender in a school-based sample of 403 9th grade (13 to 16-year-old) adolescents |
| **Study design** | Cross-sectional |
| **Study setting** | School setting |
| **Participants** | 403 |
| **Gender distribution** | 49 % males |
| **Type of social media use** | Cyberbullying and victimization |
| **How social media was assessed** | Frequency of online victimization |
| **Mental Health or well-being measure** | Depressive symptoms, suicidal ideation |
| **Type of scales used** | Cyberbullying and Victimization Survey (CBVS), Children's Depression Inventory 2nd Edition Short Version, Suicidal Ideation Questionnaire-Junior Version. |
| **Report gender differences** | Yes |
| **Main findings** | The current investigation confirms the complexity of the association between peer victimization and suicidal ideation and that depressive symptoms, as well as gender, may play a role in this complex relation |

# Study # 63: **«**Prevalence and effect of cyberbullying on children and young people: A scoping review of social media studies**»**

| **Title** | Prevalence and effect of cyberbullying on children and young people: A scoping review of social media studies |
| --- | --- |
| **Authors** | Hamm, M. P., Newton, A. S., Chisholm, A., Shulhan, J., Milne, A., Sundar, P., Ennis, H., Scott, S. D., Hartling, L. |
| **Year** | 2015 |
| **Journal name** | JAMA Pediatrics |
| **Aims** | To review existing publications that examine the health-related effects of cyberbullying via social media among children and adolescents |
| **Study design** | Scoping review |
| **Study setting** | Varied |
| **Participants** | Not mentioned |
| **Gender distribution** | Not mentioned |
| **Type of social media use** | Varied |
| **How social media was assessed** | Varied |
| **Mental Health or well-being measure** | Varied |
| **Type of scales used** | Varied |
| **Report gender differences** | NA |
| **Main findings** | There is a consistent relationship across studies between cyberbullying and depression among children and adolescents; however, the evidence of the effect of cyberbullying on other mental health conditions is inconsistent. This review provides important information that characterizes cyberbullying within the context of social media, including attributes of the recipients and perpetrators, reasons for and the nature of bullying behaviors, and how recipients react to and manage bullying behaviors. |

# Study # 64: **«**Impacts of traditional bullying and cyberbullying on the mental health of middle school and high school students**»**

| **Title** | Impacts of traditional bullying and cyberbullying on the mental health of middle school and high school students |
| --- | --- |
| **Authors** | Hase, C. N., Goldberg, S. B., Smith, D., Stuck, A., Campain, J. |
| **Year** | 2015 |
| **Journal name** | Psychology in the Schools |
| **Aims** | The current study sought to address the following questions: (1) Does cyberbullying create new victims or merely a new means of victimization? (2) Does cyberbullying uniquely contribute to negative outcomes above and beyond those of traditional bullying? |
| **Study design** | Cross-sectional |
| **Study setting** | School setting |
| **Participants** | 1225 |
| **Gender distribution** | 632 males |
| **Type of social media use** | Online bullying |
| **How social media was assessed** | Being left out, online rumours etc |
| **Mental Health or well-being measure** | Psychological symptoms |
| **Type of scales used** | Strengths and Difficulties Questionnaire, The nine-item Cyberbullying Questionnaire, California Bullying Victimization Scale |
| **Report gender differences** | Yes |
| **Main findings** | Both forms of victimization were independently associated with negative outcomes. However, when controlling for traditional bullying, cyberbullying did not remain a predictor of negative mental health outcomes. In contrast, when controlling for cyberbullying, traditional bullying remained a significant predictor of negative mental health outcomes. These results suggest that although traditional and cyber forms of bullying tend to target the same victims, traditional bullying is more uniquely associated with negative psychological outcomes |

# Study # 65: **«**Self-Harm, suicidal behaviours, and cyberbullying in children and young people: Systematic review**»**

| **Title** | Self-Harm, suicidal behaviours, and cyberbullying in children and young people: Systematic review |
| --- | --- |
| **Authors** | John, A., Glendenning, A. C., Marchant, A., Montgomery, P., Stewart, A., Wood, S., Lloyd, K., Hawton, K. |
| **Year** | 2018 |
| **Journal name** | Journal of Medical Internet Research |
| **Aims** | The aim of this study was to systematically review the current evidence examining the association between cyberbullying involvement as victim or perpetrator and self-harm and suicidal behaviors in children and young people (younger than 25 years), and where possible, to meta-analyze data on the associations. |
| **Study design** | Systematic review |
| **Study setting** | Varied |
| **Participants** | 156384 |
| **Gender distribution** | Varied |
| **Type of social media use** | Varied |
| **How social media was assessed** | Varied |
| **Mental Health or well-being measure** | Self-harm, suicidal behavior |
| **Type of scales used** | Varied |
| **Report gender differences** | Yes |
| **Main findings** | Victims of cyberbullying are at a greater risk than nonvictims of both self-harm and suicidal behaviors. To a lesser extent, perpetrators of cyberbullying are at risk of suicidal behaviors and suicidal ideation when compared with nonperpetrators |

# Study # 66: **«**Cyberbullying and adolescent well-being in England: a population-based cross-sectional study**»**

| **Title** | Cyberbullying and adolescent well-being in England: a population-based cross-sectional study |
| --- | --- |
| **Authors** | Przybylski, A. K., Bowes, L. |
| **Year** | 2017 |
| **Journal name** | The Lancet Child & Adolescent Health |
| **Aims** | We aimed to estimate the prevalence of cyberbullying and traditional bullying among adolescents in England, and assess its relative effects on mental well-being. |
| **Study design** | Cross-sectional |
| **Study setting** | School setting |
| **Participants** | 110788 |
| **Gender distribution** | 35 % males |
| **Type of social media use** | Olweus Bully/Victim Questionnaire |
| **How social media was assessed** | Mean messages, posts, web-sites |
| **Mental Health or well-being measure** | Well-being |
| **Type of scales used** | Olweus Bully/Victim Questionnaire |
| **Report gender differences** | Yes |
| **Main findings** | Traditional bullying is considerably more common among adolescents in Englandthan cyberbullying. While both forms of bullying were associated with poorer mental well-being, cyberbullying accounted for a very small share of variance after adjustment for offline bullying and other covariates. |

# Study # 67: **«**Cyberbullying, help-seeking and mental health in young Australians: implications for public health**»**

| **Title** | Cyberbullying, help-seeking and mental health in young Australians: implications for public health |
| --- | --- |
| **Authors** | Spears, B. A., Taddeo, C. M., Daly, A. L., Stretton, A., Karklins, L. T. |
| **Year** | 2015 |
| **Journal name** | International Journal of Public Health |
| **Aims** | To examine the relationship between young Australians’ cyberbullying experiences, their help-seeking practices and associated mental well-being and social connectedness, with a view to informing national health and well-being agendas. |
| **Study design** | Cross-sectional |
| **Study setting** | Home setting |
| **Participants** | 2338 |
| **Gender distribution** | 44 % males |
| **Type of social media use** | Pictures, webcam or video clips, Phone calls, Email, Chat sites, Instant messaging, e.g., MSN Messenger, Social networking sites, e.g., Facebook, Online gaming, Webpage, Twitter |
| **How social media was assessed** | Frequency of cyberbullying |
| **Mental Health or well-being measure** | General mental health, well-being, depression, anxiety, stress |
| **Type of scales used** | Mental health continuum short form, Depression Anxiety Stress Scales-21, self-developed cyberbullying questionnaire |
| **Report gender differences** | Yes |
| **Main findings** | Youth with no experience of cyberbullying had better well-being profiles and mental health overall. Conversely, cyberbully victims, had poorer well-being and mental health and tended not to engage with online support services, in spite of being more likely to be online after 11 pm. |

# Study # 68: **«**Cyberbullying: a storm in a teacup? **»**

| **Title** | Cyberbullying: A storm in a teacup? |
| --- | --- |
| **Authors** | Wolke, D., Lee, K., Guy, A. |
| **Year** | 2017 |
| **Journal name** | European Child & Adolescent Psychiatry |
| **Aims** | Our aim was to determine whether cyberbullying creates uniquely new victims, and whether it has similar impact upon psychological and behavioral outcomes for adolescents, beyond those experienced by traditional victims. |
| **Study design** | Cross-sectional |
| **Study setting** | Not mentioned |
| **Participants** | 2745 |
| **Gender distribution** | 43 % males |
| **Type of social media use** | Cyber-victimization e.g. spreading of rumours, posting pictures withour permission etc. |
| **How social media was assessed** | Frequency of cyberbullying |
| **Mental Health or well-being measure** | Psychiatric problems |
| **Type of scales used** | Bullying and Friendship Interview schedule, Strengths and Diffculties Questionnaire |
| **Report gender differences** | Yes |
| **Main findings** | Cyberbullying creates few new victims, but is mainly a new tool to harm victims already bullied by traditional means. Cyberbullying  extends the reach of bullying beyond the school  gate. Intervention strategies against cyberbullying may need  to include approaches against traditional bullying and its  root causes to be successful. |

# Study # 69: **«**Understanding adolescent students' use of facebook and their subjective wellbeing: A gender-based comparison**»**

| **Title** | Understanding adolescent students' use of facebook and their subjective wellbeing: A gender-based comparison |
| --- | --- |
| **Authors** | Lai, Hui-Min, Hsieh, Pi-Jung, Zhang, Ren-Cheng |
| **Year** | 2018 |
| **Journal name** | Behaviour & Information Technology |
| **Aims** | The current study examines the determinants of Facebook use and its relationship with adolescents’ subjective wellbeing, stratified by gender. |
| **Study design** | Cross-sectional |
| **Study setting** | School setting |
| **Participants** | 1121 |
| **Gender distribution** | 625 males |
| **Type of social media use** | Facebook use |
| **How social media was assessed** | Number of Facebook friends, amount of Facebook use |
| **Mental Health or well-being measure** | Well-being |
| **Type of scales used** | Self-developed measures |
| **Report gender differences** | Yes |
| **Main findings** | The results indicated that the total number of Facebook friends, the need to belong and the perceived waste of time were correlated with adolescents’ use of Facebook; in turn, Facebook use was positively related to the subjective wellbeing of both male and female students. Gender  differences were also observed; the need to belong and the positive relationship between Facebook use and subjective wellbeing were both stronger for male students than female students. |

# Study # 70: **«**Depressive symptoms in adolescents**»**

| **Title** | Depressive symptoms in adolescents |
| --- | --- |
| **Authors** | Wartberg, L., Kriston, L., Thomasius, R. |
| **Year** | 2018 |
| **Journal name** | Deutsches Arzteblatt International |
| **Aims** | The aims of our study were to determine: The current point prevalence of depressive symptoms among adolescents in Germany and which sociodemographic and psychosocial features are associated with depressive symptoms in adolescents. |
| **Study design** | Cross-sectional |
| **Study setting** | Home setting |
| **Participants** | 1001 |
| **Gender distribution** | 52 % males |
| **Type of social media use** | Problematic use of social media like websites of social networks or messengers, and Social Media Disorder Scale. |
| **How social media was assessed** | Article does not say |
| **Mental Health or well-being measure** | Depression, body image |
| **Type of scales used** | The validated Depression Screener for Teenagers, body image questionnaire, Social Media Disorder Scale, Internet Gaming Disorder Scale |
| **Report gender differences** | Yes |
| **Main findings** | A substantial percentage of German adolescents suffers from depressive symptoms. This study was the first to show certain associations, such as that between depressive symptoms in adolescence and the problematic use of social media in German youth. |

# Study # 71: **«**Facebook addiction and its relationship with mental health among Thai high school students**»**

| **Title** | Facebook addiction and its relationship with mental health among thai high school students |
| --- | --- |
| **Authors** | Hanprathet, N., Manwong, M., Khumsri, J., Yingyeun, R., Phanasathit, M. |
| **Year** | 2015 |
| **Journal name** | Journal of the Medical Association of Thailand |
| **Aims** | To investigate the relationship between Facebook addiction and mental health among high school students. |
| **Study design** | Cross-sectional |
| **Study setting** | School setting |
| **Participants** | 972 |
| **Gender distribution** | 37 % male |
| **Type of social media use** | Facebook use |
| **How social media was assessed** | Bergen Facebook Addiction Scale |
| **Mental Health or well-being measure** | General mental health |
| **Type of scales used** | Bergen Facebook Addiction Scale, The General Health Questionnaire |
| **Report gender differences** | Yes |
| **Main findings** | It was found that Facebook addiction among high school students could be associated with abnormal, general mental health status, somatic symptoms, anxiety and insomnia, social dysfunction, and severe depression. |

# Study # 72: **«**Facebook: Risks and opportunities in Brazilian and Portuguese youths with different levels of psychosocial adjustment**»**

| **Title** | Facebook: Risks and opportunities in Brazilian and Portuguese youths with different levels of psychosocial adjustment |
| --- | --- |
| **Authors** | Marques, T. P., Marques-Pinto, A., Alvarez, M. J., Pereira, C. R. |
| **Year** | 2018 |
| **Journal name** | The Spanish Journal of Psychology |
| **Aims** | This study aimed to assess the risks and opportunities associated with Facebook usage and to explore the moderating role of psychosocial (mal)adjustment, nationality and age in these relationships. |
| **Study design** | Cross-sectional |
| **Study setting** | Home setting |
| **Participants** | 952 |
| **Gender distribution** | 381 males |
| **Type of social media use** | Facebook use |
| **How social media was assessed** | Risks, opportunities and usage habits of Facebook |
| **Mental Health or well-being measure** | Psychosocial adjustment |
| **Type of scales used** | Assessment of Risks and Opportunities in Young Users of Facebook Scale, Social and Emotional Loneliness Scale, Social Anxiety Scale for Adolescents |
| **Report gender differences** | Yes |
| **Main findings** | The results of this study should be analyzed in more detail in the future by collecting a more homogeneous sample, from both countries, in terms of age, and by further adding a qualitative approach in order to clarify the cultural aspects of the differences. On a final note, given the increasing number of children and adolescents who create profiles on this social network, in addition to the amount of time they spend on it, there will certainly be some impact, not only on their daily lives, but also on their development, and these effects should be the focus of future studies. |

# Study # 73: **«**The association between social networking sites and alcohol abuse among Belgian adolescents: The role of attitudes and social norms**»**

| **Title** | The association between social networking sites and alcohol abuse among Belgian adolescents: The role of attitudes and social norms |
| --- | --- |
| **Authors** | Geusens, F., Beullens, K. |
| **Year** | 2018 |
| **Journal name** | Journal of Media Psychology |
| **Aims** | The current cross-sectional study (N = 3,133) aims to explore the relation between being exposed to and displaying alcohol-related content on social networking sites (SNSs) with alcohol abuse among adolescents aged 16–20 years. |
| **Study design** | Cross-sectional |
| **Study setting** | School setting |
| **Participants** | 3133 |
| **Gender distribution** | 51 % males |
| **Type of social media use** | Social network sites |
| **How social media was assessed** | Exposure to or display of alcohol-related content on SNSs |
| **Mental Health or well-being measure** | Alcohol use, alcohol attitude |
| **Type of scales used** | Alcohol Use Disorders Identification Test scale, self-developed measures of alcohol displaying or exposure. |
| **Report gender differences** | Yes |
| **Main findings** | Furthermore, the associations of both exposure to and displaying alcohol-related content on SNS with alcohol abuse are mediated through perceived social norms of friends and attitudes toward excessive alcohol consumption. Yet, whereas alcohol-related attitudes and social norms seemed to be equally important mediators in the relation between exposure to alcohol-related content on SNS and alcohol abuse, the association between displaying alcohol-related content on SNS and alcohol abuse seemed to be mediated predominantly through a change in alcohol-related attitudes. Overall, this study sheds more light on the complex relations between SNS use and risky drinking behavior among adolescents |

# Study # 74: **«**Sex differences in the association between cyberbullying victimization and mental health, substance use, and suicidal ideation in adolescents**»**

| **Title** | Sex differences in the association between cyberbullying victimization and mental health, substance use, and suicidal ideation in adolescents |
| --- | --- |
| **Authors** | Kim, S., Kimber, M., Boyle, M. H., & Georgiades, K |
| **Year** | 2019 |
| **Journal name** | The Canadian Journal of Psychiatry |
| **Aims** | To examine sex differences in the association between cyberbullying victimization and mental health (psychological distress and delinquency), substance use-related outcomes (drug and tobacco use, binge drinking), and suicide ideation among adolescents |
| **Study design** | Cross-sectional |
| **Study setting** | School setting |
| **Participants** | 4940 |
| **Gender distribution** | 43 % males |
| **Type of social media use** | Cyberbullying |
| **How social media was assessed** | Bullying through internet |
| **Mental Health or well-being measure** | Suicidal ideation, psychological distress, drug use, alcohol use |
| **Type of scales used** | Self-developed measures |
| **Report gender differences** | Yes |
| **Main findings** | Adolescents exposed to cyberbullying victimization demonstrate an increased odds of poorer mental health, substance use outcomes, and suicide ideation. The current study reveals increased risk among female adolescents as compared with male adolescents. These findings lend support for the need to develop and evaluate targeted preventative interventions specifically tailored for female and male adolescents. |

# Study # 75: **«**Participation with alcohol marketing and user-created promotion on social media, and the association with higher-risk alcohol consumption and brand identification among adolescents in the UK**»**

| **Title** | Participation with alcohol marketing and user-created promotion on social media, and the association with higher-risk alcohol consumption and brand identification among adolescents in the UK |
| --- | --- |
| **Authors** | Critchlow, N., MacKintosh, A. M., Hooper, L.,  Thomas, C., Vohra, J. |
| **Year** | 2019 |
| **Journal name** | Addiction Research & Theory |
| **Aims** | To explore participation with alcohol marketing (i.e. commenting on brand statuses) and user-created promotion on social media (i.e. photos of peers drinking) by young people in the UK, and what association this has with higher-risk consumption and brand identification. |
| **Study design** | Cross-sectional |
| **Study setting** | Home setting |
| **Participants** | 3399 |
| **Gender distribution** | 49 % males |
| **Type of social media use** | 10 most used social media apps |
| **How social media was assessed** | Social media activities towards alcohol online |
| **Mental Health or well-being measure** | Alcohol use and higher risk drinking |
| **Type of scales used** | Weekly social media use and likes, share, follow, searches and participation in competitions |
| **Report gender differences** | Yes |
| **Main findings** | Social media provides opportunities for adolescents to participate with commercial marketing and user-created promotion and this is associated with higher-risk consumption and brand identification. |

# Study # 76: **«**Motivational processes and dysfunctional mechanisms of social media use among adolescents: A qualitative focus group study**»**

| **Title** | Motivational processes and dysfunctional mechanisms of social media use among adolescents: A qualitative focus group study |
| --- | --- |
| **Authors** | Throuvala, M. A., Griffiths, M. D., Rennoldson, M., Kuss, D. J. |
| **Year** | 2018 |
| **Journal name** | Computers in Human Behavior |
| **Aims** | It was therefore, hypothesized that adolescent motivational factors would be under-pinned by (i) dysfunctional mechanisms of fear of missing out (FoMO), (ii) nomophobia, and (iii) peerpressure for constant presence and interactivity online and (iv) need for checking. This hypothesis is extending research evidence suggesting that FoMO mediates need satisfaction, mood and engagement, which may lead to a cyclical process reinforcing use. |
| **Study design** | Focus group interviews |
| **Study setting** | Not mentioned |
| **Participants** | 42 |
| **Gender distribution** | 52 % males |
| **Type of social media use** | Varied |
| **How social media was assessed** | Varied |
| **Mental Health or well-being measure** | Varied |
| **Type of scales used** | Interview |
| **Report gender differences** | Yes |
| **Main findings** | These findings shed light on cognitive-emotive aspects that may be implicated in problematic use and may inform interventions targeting excessive or problematic screen time and specific social media use aspects that merit scientific attention. |

# Study # 77: **«**Internalizing symptoms and externalizing problems: Risk factors for or consequences of cyber victimization? **»**

| **Title** | Internalizing symptoms and externalizing problems: Risk factors for or consequences of cyber victimization? |
| --- | --- |
| **Authors** | Holfeld, B., Mishna, F. |
| **Year** | 2018 |
| **Journal name** | Journal of Youth and Adolescence |
| **Aims** | In the current study, traditional victimization was controlled to examine the longitudinal sequences of association between cyber victimization and internalizing symptoms, and between cyber victimization and externalizing problems for adolescent boys and girls |
| **Study design** | Longitudinal |
| **Study setting** | School setting |
| **Participants** | 510 |
| **Gender distribution** | 38 % males |
| **Type of social media use** | Cyberbullying |
| **How social media was assessed** | Cybervictimization |
| **Mental Health or well-being measure** | Internalizing problems, externalizing problems |
| **Type of scales used** | Self-developed questionnaire |
| **Report gender differences** | Yes |
| **Main findings** | Findings from longitudinal path models suggest that internalizing symptoms and externalizing problems respectively were associated with increases in experiences of cyber victimization (beyond the effect of traditional victimization) both within and across time, particularly for adolescent girls |

# Study # 78: **«**Impacts of the use of social network sites on users' psychological well‐being: A systematic review**»**

| **Title** | Impacts of the use of social network sites on users' psychological well‐being: A systematic review |
| --- | --- |
| **Authors** | Erfani, S. S., Abedin, B. |
| **Year** | 2018 |
| **Journal name** | Journal of the Association for Information Science and Technology |
| **Aims** | As Social Network Sites (SNSs) are increasingly becoming part of people’s everyday lives, the implications of their use need to be investigated and understood. We conducted a systematic literature review to lay the groundwork for understanding the relationship between SNS use and users’ psychological well-being and for devising strategies for taking advantage of this relationship |
| **Study design** | Systematic review |
| **Study setting** | Varied |
| **Participants** | Not mentioned |
| **Gender distribution** | Not mentioned |
| **Type of social media use** | General social networks use |
| **How social media was assessed** | Varied |
| **Mental Health or well-being measure** | Psychological well-being |
| **Type of scales used** | Varied |
| **Report gender differences** | No |
| **Main findings** | Findings revealed that the use of SNSs is both positively and negatively related to users’ psychological well-being |

# Study # 79: **«**“I don’t need people to tell me I’m pretty on social media:” A qualitative study of social media and body image in early adolescent girls**»**

| **Title** | “I don’t need people to tell me I’m pretty on social media:” A qualitative study of social media and body image in early adolescent girls |
| --- | --- |
| **Authors** | Burnette, C. B., Kwitowski, M. A., Mazzeo, S. E. |
| **Year** | 2017 |
| **Journal name** | Body Image |
| **Aims** | The primary aim of this study was to examine the nature and extent of an early adolescent femail sample’s engagement with social media, and their perceptions of its impact on body image. |
| **Study design** | Focus group interviews |
| **Study setting** | Not mentioned |
| **Participants** | 38 |
| **Gender distribution** | 0 % males |
| **Type of social media use** | General social media use |
| **How social media was assessed** | Varied |
| **Mental Health or well-being measure** | Body image |
| **Type of scales used** | Varied |
| **Report gender differences** | NA |
| **Main findings** | In this sample, social media use was high. Girls endorsed some appearance concerns and social comparison, particularly with peers. However, they displayed high media literacy, appreciation of differences, and confidence, strategies that appeared helpful in mitigating the potential negative association between social media exposure and body image. |
