# Supplementary material for: Social Media Use and Mental Health and Well-Being Among Adolescents – A Scoping Review
Source: Front Psychol. 2020 Aug 14;11:1949. doi: 10.3389/fpsyg.2020.01949 (PMC7457037; doi:10.3389/fpsyg.2020.01949)
Supplement: Supplementary file 3 [file Data_Sheet_1.docx]

**Database: Embase 1974 to 2019 March 14**

**Date**: 15.03.2019

**Identified studies**: 2410

| 1 | exp Adolescence/ or exp Adolescent/ or juvenile/ or pediatrics/ or student/ | 1601435 |
| --- | --- | --- |
| 2 | (adolescen* or pediatric* or paediatric* or preadolescent* or preteen or tween or juvenil* or under?age* or teen? or teenager? or minor* or pubescen* or young people or young person* or youth* or student*).tw,kw. | 1567364 |
| 3 | 1 or 2 | 2606283 |
| 4 | Social Media/ or Video Game/ or video game console/ | 16745 |
| 5 | (Social Media or Social Medium or Online Social Network or Internet communication or communicating online or Internet group? or twitter or snapchat or facebook or messenger or youtube or Instagram or tumblr or reddit or pinterest or blog? or Video game* or Computer game* or Videogame* or Computergame* or PlayStation or Xbox or Nintendo or cyberbull* or cyber bull* or cyberharassment or cyber harassment or internet bully* or internet bullies or internet harassment or net bully* or nett bullies or net harassment or digital bully* or digital bullies or digital harassment or electronic bully* or electronic bullies or electronic harassment or webbull* or web bully* or web bullies or web harassment or online bull* or online harassment or chat bully* or chat bullies or chat harassment or cybervictim* or cyber victim* or trolling).tw,kw. | 91963 |
| 6 | 4 or 5 | 97272 |
| 7 | "psychological and psychiatric procedures"/ or exp mental health/ or exp mental health care/ or exp psychiatric diagnosis/ or exp "psychiatric theories and schools"/ or psychiatric treatment/ or convulsive therapy/ or counter transference/ or crisis intervention/ or electroconvulsive therapy/ or hypnosis/ or narcotherapy/ or observation/ or psychoanalysis/ or psychopharmacotherapy/ or exp psychotherapy/ or suggestion/ or transference/ or exp psychodynamics/ or exp psychiatry/ or exp mental disease/ or behavioral science/ or parapsychology/ or exp psychology/ or exp psychomotor disorder/ or psychiatric department/ or exp psychiatric nursing/ or Recidivism/ or anxiety/ or exp sleep disorder/ or wellbeing/ or psychological well-being/ or "quality of life"/ or happiness/ or welfare/ or human relation/ or friendship/ or friend/ or social support/ | 3131625 |
| 8 | (mental or psychiatric* or psychologic* or mood or dissociat* or Neurotic or sleep or sleeping or Insanit* or psychopatholog* or depression? or depressive or depressed or anxiety or anxieties or bipolar* or Schizophren* or psychotic* or psychosis* or psychoses* or bipolar or mania or manic or anorexi* or bulimi* or borderline or "self injur*" or "self harm" or Suicide or alcohol or ((eating or personality or panic* or phobic* or traumatic stress or amnesia* or amnestic* or "obsessive compulsive") adj disorder?) or ((multiple or dual) adj (personalit* or identit*)) or wellbeing or well being or Life Quality or "Quality of Life" or Thrive or Thriving or Contentment or Happiness or welfare or friend? or Friendship? or human relation? or interhuman relation? or Interpersonal Relationship? or Social Support).tw,kw. | 2629322 |
| 9 | 7 or 8 | 4160771 |
| 10 | 3 and 6 and 9 | 5411 |
| 11 | limit 10 to (conference abstracts or embase) | 3564 |
| 12 | limit 11 to yr="2014 -Current" | 2410 |
